# Essential Metalloelement Chelates Facilitate Repair of Radiation Injury

**DOI:** 10.1155/MBD.2001.215

**Published:** 2001

**Authors:** John R. J. Sorenson, Lee S. F. Soderberg, Louis W. Chang, Richard B. Walker

**Affiliations:** 1 University of Arkansas Medical Sciences Campus, Little Rock Division of Medicinal Chemistry Department of Pharmaceutical Sciences, College of Pharmacy Arkansas 72205 USA; 2 University of Arkansas Medical Sciences Campus, Little-Rock Department of Microbiology and Immunology College of Medicine Arkansas 72205 USA; 3 National Health Research Institutes Division of Environmental Health and Occupational Medicine Taiwan China; 4 University of Arkansas at Pine Bluff Department of Chemistry and Physics Pine Bluff Arkansas 71611 USA

## Abstract

Treatment with essential metalloelement (Cu, Fe, Mn, and Zn) chelates or combinations of them **before** and/or **after** radiation injury is a useful approach to overcoming radiation injury. No other agents are known to increase survival when they are used to treat **after** irradiation, in a radiorecovery treatment paradigm. These chelates may be useful in facilitating *de novo* syntheses of essential metalloelement-dependent enzymes required to repair radiation injury. Reports of radioprotection, which involves treatment **before** irradiation, with calcium-channel blockers, acyl Melatonin homologs, and substituted anilines, which may serve as chelating agents after biochemical modification *in vivo*, as well as Curcumin, which is a chelating agent, have been included in this review. These inclusions are intended to suggest additional approaches to combination treatments that may be useful in facilitating radiation recovery. These approaches to radioprotection and radiorecovery offer promise in facilitating recovery from radiation-induced injury experienced by patients undergoing radiotherapy for neoplastic disease and by individuals who experience environmental, occupational, or accidental exposure to ultraviolet, x-ray, or γ-ray radiation. Since there are no existing treatments of radiation-injury intended to facilitate tissue repair, studies of essential metalloelement chelates and combinations of them, as well as combinations of them with existing organic radioprotectants, seem worthwhile.


### ESSENTIAL METALLOELEMENT CHELATES FACILITATE REPAIR OF RADIATION INJURY

John R. J. Sorenson*[1a], Lee S. F. Soderberg[1b], Louis W. Chang[2], and Richard B. Walker[3]

[1] University of Arkansas, Medical Sciences Campus, Little Rock, Arkansas 72205, USA
[a] Division of Medicinal Chemistry, Department of Pharmaceutical Sciences, College of Pharmacy
<sorensonjohnrj@uams.edu> [b] Department of Microbiology and Immunology, College of Medicine
[2] National Health Research Institutes, Division of Environmental Health and Occupational Medicine Taiwan, Republic of China,
[3] University of Arkansas at Pine Bluff, Department of Chemistry and Physics
Pine Bluff, Arkansas 71611, USA

### ABSTRACT

Treatment with essential metalloelement (Cu, Fe, Mn, and Zn) chelates or combinations of them **before** and/or **after** radiation injury is a useful approach to overcoming radiation injury. No other agents are known to increase survival when they are used to treat **after** irradiation, in a radiorecovery treatment paradigm. These chelates may be useful in facilitating *de novo* syntheses of essential metalloelement-dependent enzymes required to repair radiation injury. Reports of radioprotection, which involves treatment **before** irradiation, with calcium-channel blockers, acyl Melatonin homologs, and substituted anilines, which may serve as chelating agents after biochemical modification *in vivo*, as well as Curcumin, which is a chelating agent, have been included in this review. These inclusions are intended to suggest additional approaches to combination treatments that may be useful in facilitating radiation recovery. These approaches to radioprotection and radiorecovery offer promise in facilitating recovery from radiation-induced injury experienced by patients undergoing radiotherapy for neoplastic disease and by individuals who experience environmental, occupational, or accidental exposure to ultraviolet, x-ray, or γ-ray radiation. Since there are no existing treatments of radiation-injury intended to facilitate tissue repair, studies of essential metalloelement chelates and combinations of them, as well as combinations of them with existing organic radioprotectants, seem worthwhile.

### INTRODUCTION

### Essentiality and Speciation of Cu, Fe, Mn, and Zn In Vivo

Copper, iron, manganese, and zinc are essential metalloelements like sodium, potassium, calcium, magnesium, chromium, cobalt, and vanadium. These essential metalloelements as well as essential amino acids, essential fatty acids, and essential cofactors (vitamins) are required by all cells for normal metabolic processes but cannot be synthesized *de novo* and dietary intake and absorption are required to obtain them. Amounts of Cu, Fe, Mn, and Zn found in body tissues and fluids [1,2] correlate with the number and kind of metabolic processes requiring them. However, there may be decreases in tissue content due to inadequate dietary intake and absorption associated with soil depletion, socioeconomic circumstances, and aging-related declines in consumption.

Ionic forms of these metalloelements have particularly high affinities for organic ligands found in biological systems and rapidly undergo bonding interactions to form complexes or chelates in biological systems. Calculated amounts of ionic Cu ($10^{-18}$M), Fe ($10^{-23}$M), Mn ($10^{-12}$M), and Zn ($10^{-9}$M) in plasma [3] are extremely small and not measurable with existing equipment. Concentrations of these ionically bonded essential metalloelements are likely to be even smaller in solid tissues, which contain many more bonding ligands. Consequently, measured tissue Cu, Fe, Mn, and Zn contents reflect tissue contents of chelates. These are primarily metalloelement-dependent enzymes (Table 1), proteins such as Hemoglobin, Ferritin, Metallothioneines (MTs), $\beta_2$-Macroglobulins, and Transferrin, as well as RNA and DNA chelates, small molecular mass amino acid, carboxylic acid, phosphate, amine, diamine, and thiol chelates and various small molecular mass peptide chelates [4 and cited refs.]. Ionically bonded metalloelement compounds will immediately form coordinate-covalent bonded chelates in biological systems as a result of bonding interactions with components of these systems capable of these bonding interactions. To study effects of essential metalloelements in a biological system it is most inappropriate to use ionically bonded forms of a particular metalloelement and most appropriate to use relevant chelates, which have particularly important roles in mediating biochemical repair of radiation injury *via* ligand exchange processes.

Table I. Established and recently suggested Cu, Fe, Mn, and Zn dependent mammalian enzymes.

| Cu-dependent enzymes [4-19] | Function |
|---|---|
| Cytochrome c Oxidase | Reduction of oxygen |
| Extracellular and Cytosolic Cu-Dependent and Zn-Modulated Superoxide Dismutases ($Cu_2Zn_2SOD$) | Superoxide disproportionation |
| Tyrosinase | Synthesis of dihydroxyphenylalanine |
| Dopamine-ß-Mono-Oxygenase | Synthesis of norepinephrine and epinephrine |
| Neurocuprein | Synthesis of norepinephrine and epinephrine |
| Amine Oxidases | Metabolism of primary amines |
| Factors V and VIII | Blood clotting |
| Ceruloplasmin | Cu transport, mobilization of stored Fe, and angiogenesis, down-regulation of nitric oxide synthase (NOS) |
| α-Amidating Mono-Oxygenases | Synthesis of neuroendocrine hormones including: gastrin, choleocystokinin, melanocyte stimulating hormone, calcitonin, vasopressin, secretin, and some enkephalins |
| Procollagen and Proelastin Peptidyllysyl Oxidases | Collagen and elastin cross-linking |
| Prion Protein | Normal metalloglycoprotein located on the extracellular surface of many cell types of unknown function |
| Methionine Synthase | Synthesis of methione from homocysteine |
| Amyloid Precursor Protein | May have a role in decreasing amyloid $A^{\beta}$ protein synthesis and increasing secretion of amyloid precursor protein |
| Plasma Pituitary Adenylate Cyclase-Activating Polypeptide | Activates Adenylyl Cyclase and Modulates inducible Nitric Oxide Synthase (NOS) |
| CCS | Cu Chaperone for $Cu_2Zn_2SOD$ |
| Other possible Cu-dependent enzymes [20-24] | |
| Adenylate Cyclase | Synthesis of c-AMP |
| Guanylate Cyclase | Synthesis of c-GMP |
| Lipolytic Protein | Lipolysis |
| ACE1 and CUP2 | Metallothioneine gene transcription regulatory proteins |
| Iron-dependent enzymes [5,25] | |
| Electron Transport Cytochromes including Cytochrome c Oxidase | Oxidation-Reduction |
| Catalase | Disproportionation of hydrogen peroxide |
| P-450s | Activation of oxygen |
| Lipoxygenase | Conversion of arachadonic acid to 5-HPETE and 5-HETE |
| Cyclo-Oxygenase | Conversion of aracadonic acid to $PGG_2$ |
| Xanthine Oxidase | Purine metabolism |
| Peptidylprolyl and Peptidyllysyl Oxidases | Hydroxylation of procollagen and proelastin |
| Catechol Dioxygenases | Oxidative opening of catechol ring systems |
| Nitric Oxide (Nitrogen Monoxide) Synthase | Synthesis of nitrogen monoxide |

| | |
|---|---|
| Manganese-dependent enzymes [5,26-32] | |
| Mn-Dependent SOD | Disproportionation of superoxide |
| Arginase | Syntheses of urea and ornithine |
| Pyruvate Carboxylase | Synthesis of oxaloacetate |
| Pseudocatalase | Disproportionation of $H_2O_2$ |
| Calmodulin-Dependent Protein | Phosphate ester hydrolysis |
| Phosphatase | |
| α-Isopropylmalate Synthetase | Synthesis of α-isopropylmalate |
| A Brain Adenylate Cyclase | Synthesis of c-AMP |
| Catechol-O-Methyltransferase | Synthesis of catechol methyl ether |
| Farnesyl Pyrophosphate Synthetase | Synthesis of farnesyl pyrophosphate |
| Glycosyl Synthetase | Synthesis of glycosyl-phosphonucleotides |
| Extradiol-Cleaving Dioxygenase | Insertion of dioxygen |
| A Ganglioside Galactosyl- | Galactose transfer |
| Saccharide Polymerases | Synthesis of polymeric carbohydrates |
| Glycosyltransferases transferase | Synthesis of glycosides |
| A Galactosylhydroxylysyl | Glycosaminoglycan and glycoprotein |
| connective Glucosyltransferase | tissue component syntheses |
| Phosphatases | Phosphate ester hydrolysis |
| Restriction Endonucleases | DNA recognition and cleavage |
| Xylose Isomerase | Isomerization of xylose |
| Catalases | Disproportionation of hydrogen peroxide |
| Extradiol-Cleaving Catechol Dioxygenases | Oxidative opening of catechol ring systems |
| Zinc-dependent enzymes [5,33-36] | |
| $Cu_2Zn_2SOD$ | Disproportionation of superoxide |
| Aminopeptidase | Protein hydrolysis |
| Aldehyde Hydrase | Aldehyde hydration |
| Esterase | Ester hydrolysis |
| Methylmalonyl-Oxaloacetate | Transcarboxylation |
| Transcarboxylase | |
| Carboxypeptidases A and B | Protein hydrolysis |
| NAD-Dependent Dehydrogenases | Oxidations |
| Carbonic Anhydrase | Dehydration of carbonic acid |
| α-Hydroxyacid Dehydrgenase | Oxidation of α-hydroxy acids |
| Alkaline Phosphatase | Phosphorylation |
| Purine and Pyrimidine | Phosphorylation of nucleosides |
| Nucleoside Kinases | |
| DNA Polymerase and Gyrase | DNA synthesis |
| "Zinc-Finger" proteins | Transcription regulating proteins |
| Matrix Metalloproteinases | Endopeptidases required for repair, maintenance, and function of extracellular connective tissue components and wound repair |
| Metal-Responsive Transcription Factor-1 | Metalloelement transcription factor, a Zn-finger regulatory protein required for cellular response in metalloelement uptake |

**Essential Metalloelement Metabolism**

Ingested foods and beverages contain these essential metalloelements in chelated forms, which may yield other chelates as a result of ligand exchange in the digest or following absorption [4]. However, some of the absorbed chelates may exist in the digest and in blood as the original chelate or a more stable ternary chelate. Absorbed metalloelement chelates undergo systemic circulation to all tissues and utilization by all cells following ligand exchange with small molecular mass ligands, apoproteins, and apoenzymes to form metalloproteins and metalloenzymes in *de novo* syntheses. These metalloelements are stored in the appropriate tissues as MTs or Fe-Ferritin, or they are excreted in the event tissue needs have been met and stores replenished [4]. There are no inducible excessive storage diseases known in normal individuals. However, there must be upper limits dictated by physiological requirements.

Stored essential metalloelements are released as chelates *via* ligand exchange to meet normal metabolic needs. This homeostatic release of relatively small amounts of essential metalloelement chelates meets normal physiologic requirements. Release of larger quantities in a pronounced mobilization of these metalloelements is a feature of interleukin-1 (IL-1) and other cytokine mediated acute and chronic responses to many disease states [4 and cited refs].

It is likely that the magnitude of these responses is adequate in normal well-nourished individuals and allows them to overcome their disease. However, it is now generally recognized that dietary intakes of Cu, Fe, Zn [37,38], and most likely Mn are less than recommended daily intakes for the U.S. population and may be no better for individuals living in other developed countries. Individuals who have depleted stores of these essential metalloelements, such as chronic disease patients, may overcome pathology associated with their disease state, which always involves inflammation, more slowly or not at all. The degree of radiation injury and the nutritional state of health of an individual may determine whether or not an individual will be able to overcome metalloelement-dependent repairable radiation injury. Treatment with essential metalloelement chelates either **before** and/or **after** radiation injury may support these essential metalloelement-dependent responses or facilitate recovery regardless of the state of nutriture.

**Chemical Consequences of Ionizing Radiation**

Since water represents 70% of the chemical composition of the adult body (90% of the infant body), its chemical transformation by ionizing radiation merits serious consideration with regard to chemical consequences of ionizing radiation. Ionizing radiolysis of water is well understood and produces very reactive aquated electrons, monoatomic hydrogen atoms, hydroxyl radical, hydrogen peroxide, and protonated water as well as superoxide ($O_2^-$) and hydroperoxyl radical in the presence of oxygen. Hydroperoxyl radical, hydroxyl radical, monoatomic hydrogen, and aquated electrons have very short half-lives of the order of milliseconds and consequently react rapidly with cellular components in reduction, oxidation, initiation, insertion, propagation, and addition reactions causing loss of function and the need for biochemical replacement and/or repair. Ionizing radiation can also impart sufficient energy to all biochemicals to cause homolyltic bond breaking and produce all conceivable organic radicals in considering C-C, C-N, C-O, C-H, P-O, S-O, etcetera bond homolyses. These radicals will undergo the above listed radical reactions to cause further destruction and the need for replacement and/or repair. A third consequence of ionizing radiation is homolytic or heterolytic bond breaking of coordinate-covalent bonded metalloelements. These are the weakest bonds in biochemical molecules and potential sites of greatest damage, which may be most in need of replacement and/or repair since many repair enzymes are metalloelement-dependent.

Recognizing that loss of essential metalloelement-dependent enzyme activity may at least partially account for lethality of ionizing radiation and that Cu-, Fe-, Mn-, and Zn-dependent enzymes have roles in protecting against accumulation of $O_2^-$ as well as facilitating repair [39] may explain the radiation protection and radiation recovery activities of Cu, Fe, Mn, and Zn compounds. It is suggested that the IL-1 mediated redistributions of essential metalloelements have a general role in responding to radiation injury. These redistributions may also account for subsequent *de novo* synthesis of metalloelement-dependent enzymes required for biochemical repair and replacement of cellular and extracellular components needed for recovery from radiolytic damage.

*De novo* syntheses of metalloelement-dependent enzymes required for utilization of oxygen and prevention of $O_2^-$ accumulation as well as tissue repair processes including metalloelement-dependent DNA and RNA repair are key to the hypothesis that essential metalloelement chelates decrease and/or facilitate recovery from radiation-induced pathology. A widely held understanding is that $O_2^-$ accumulates in mammalian cellular and extracellular spaces due to the lack of normal concentrations of Cu and Zn-or Mn-dependent superoxide dismutases (SODs), inappropriate reduction of $O_2$ leading to relatively large steady-state concentrations of $O_2^-$, or inappropriate release of $O_2^-$ from oxygen activating centers. Increasing synthesis of $O_2^-$ as the result of the conversion of Xanthine Dehydrogenase, which does not synthesize $O_2^-$, to Xanthine Oxidase which does synthesize $O_2^-$ has been recently demonstrated in the liver of 3 to 9 Gray (Gy) irradiated mice [40]. More reactive oxygen-radicals derived from $O_2^-$ include: singlet oxygen and hydrogen peroxide, produced as a result of acid-dependent self disproportionation or water-catalyzed disproportionation of $O_2^-$, hydroxyl radical, and hydroperoxyl radical [41,42]. Facilitated *de novo* syntheses of $Cu_2Zn_2SODs$ and catalase and decreasing or preventing formation of these oxygen-radicals may account for some of the recovery from pathological changes associated with irradiation, which cause systemic inflammatory disease and immuno-incompetency in the case of whole-body irradiation, and focal inflammatory disease associated with local irradiation.

**Radiation-Induced Tissue Injury**

Ionizing radiation is known to cause a variety of tissue injuries at all levels of biochemical systems. Acutely toxic radiation syndromes are well known in terms of doses of radiation and clinical courses of these syndromes [43]. Doses of radiation that are less than those that cause death in the short term as well as ultraviolet irradiation cause a variety of disease states depending upon the biochemical injury of the affected tissues. Consequences of ionizing and ultraviolet irradiation are a matter of current concern. Radiation risk of fatal malignancy associated with commonly performed coronary angiography was found to be around 1 in 6,000 with a risk of fatal lung cancer of about 1 in 8,000, an increase of 0.017% above the normal incidence for the general population [44]. Longer and more complicated procedures carry a greater risk consistent with the well known phenomenon of radiation-induced carcinogenesis [45]. Radiation-Induced skin injury is also an unwanted consequence of this procedure and more so for all *ex vivo* irradiations of neoplastic tumors [44].

Adult CF male rats x-irradiated with 0.015 centiGy $sec^{-1}$ $day^{-1}$ for 7 days followed by 7 days rest for either 3 months or 6 months showed duration related patchy skin hyalinization, which became completely hyalinized during the succeeding 6 months without irradiation [46]. The concentration of Zn in skin of these rats remained normal in both irradiated groups but by the end of 1 yr it had significantly ($P < 0.001$) decreased to 13 μg $gm^{-1}$ of tissue from 19 μg $gm^{-1}$. Skin Fe was elevated in both irradiated groups and the elevation increased from 100 μg $gm^{-1}$ of tissue with 3 mo irradiation to 140 μg $gm^{-1}$ after 6 mo irradiation versus 68 μg $gm^{-1}$ for control skin. There was a further relative increase in skin Fe 1 yr after irradiation to 129 μg $gm^{-1}$ of tissue compared to 65 μg $gm^{-1}$ for control skin ($P< 0.001$). It was concluded that chronic exposure to low dose x-irradiation possibly causes a redistribution of Zn and Fe with concomitant hyalinization of collagen. The observed skin collagen pathology and altered essential metalloelement contents were interpreted as results of chronic inflammation due to chronic irradiation [46].

Recent studies of pregnant women who resided in a heavily exposed area of the Republic of Belarus following the Chernobyl catastrophe appear to be at risk for development of toxemia, renal insufficiency, and anemia. Neonates born in heavily contaminated areas were found to be at risk for development of anemia, congenital malformations, and perinatal death. Decreased concentrations of erythrocyte Cu and Zn were documented for these neonates. Diminished white blood cell, T-helper cell, and T4 cell counts were found for neonates from areas of heavy exposure [47].

**Radiation Protection and Recovery with Copper Chelates.**

Small non-toxic doses of $Cu(II)_2(3,5\text{-}DIPS)_4$ ranging form 5 to 80 μmol $kg^{-1}$ of body mass increased survival of lethally irradiated mice treated orally or subcutaneously (sc) **before** irradiation, while smaller doses ranging down to 2.5 μmol $Cu(II)_2(3,5\text{-}DIPS)_4$ $kg^{-1}$ given sc 3 hr **after** irradiation, the radiation recovery paradigm, also increased survival [48 and cited refs.]. In a recent radiorecovery study, doses of 5, 10, or 20, μmol $Cu(II)_2(3,5\text{-}DIPS)_4$ $kg^{-1}$ given 3 hr **after** $LD_{50/30}$ (8 Gy, 1.4 Gy $min^{-1}$) irradiation produced survivals of 52%, 60%, or 52%, increases of 63%, 88% ($P < 0.05$), or 63%, respectively, compared to vehicle-treated control mice, and also facilitated recovery of radiation-induced loss of body mass ($P < 0.05$) and recovery of radiation-induced loss of physical activity (locomotion) ($P \leq 0.001$) [49].

Doses of 5, 10, or 20 μmol $Cu(II)Cl_2$, or 5, 10, or 20 μmol $Cu(II)_2(3,5\text{-}DIPS)_4$ $kg^{-1}$ administered sc 3 hr **after** $LD_{50/30}$ whole-body irradiation facilitated recovery of radiation-induced systemic inflammatory disease, recovery of lost body mass, and increased survival. Treatment with 5, 10, or 20 μmol $Cu(II)Cl_2$ $kg^{-1}$ produced 7%, 21,% or 29% increases in survival, respectively, compared to the vehicle-treated group, which is consistent with the activity reported by others [see 48]. However, treatment with 5, 10, or 20 μmol $Cu(II)_2(3,5\text{-}DIPS)_4$ $kg^{-1}$ produced increases of 44%, 67%, or 44% survival, respectively, compared to the vehicle-treated group. The recovery of radiation-induced loss in body mass and increased survival of mice treated with either $Cu(II)Cl_2$ or $Cu(II)_2(3,5\text{-}DIPS)_4$ document that both Cu compounds are effective radiorecovery agents. However $Cu(II)_2(3,5\text{-}DIPS)_4$, the more bioavailable form of Cu is more effective than $Cu(II)Cl_2$ [50].

All radiation protection and radiation recovery doses of $Cu(II)_2(3,5\text{-}DIPS)_4$ $Cu(II)_2(3,5\text{-}DIPS)_4$ are non-toxic and ranged from 1/5 to 1/100 of an acutely toxic dose ($LD_{50/7}$) for male or female mice [see 48]. The $LD_{50/7}$, acute toxicity, of $Cu(II)_2(3,5\text{-}DIPS)_4$ in female mice and rats, 261±36 μmol $kg^{-1}$, was much higher than observed for male mice and rats, 91±13 μmol $kg^{-1}$. Acutely lethal doses of $Cu(II)_2(3,5\text{-}DIPS)_4$ produce marked CNS depression due to loss of parasympathetic regulation of cardiac and pulmonary function. Smaller doses produce short duration hypnotic or sedative effects. The small doses of Cu(II)2(3,5-DIPS4 exhibiting radioprotectant and radiorecovery activity do not produce any of these CNS depressant effects. Of perhaps still greater interest with regard to many studies of supposed "Cu-mediated toxicity" as having a role in an "oxidative etiology", suggested to account for pathology of many disease states, is the recent report pointing out that these studies were seriously flawed [51]. The same increase in 532 nm absorbance measured following the addition of thiobarbituric acid to disease-related biological systems to which ionically bonded forms of Cu had been added to "catalyze oxidations", was observed following addition of ionically bonded Cu to aqueous solutions of only thiobarbituric acid. The linear increase in this 532 nm absorbance was related to the increase in Cu-thiobarbiturate chelate(s)formation.

Initial studies involving treatment of $LD_{100/50}$ irradiated mice **after** irradiation employed a dose of 80 μmol $Cu(II)_2(3,5\text{-}DIPS)_4$ $kg^{-1}$ which was found to be effective in increasing survival when given **before** irradiation. However this dose given **after** irradiation caused acute lethality, all of these treated mice died **before** the vehicle-treated mice (non-published). The rationale for this observation is that treatment with this large dose of $Cu(II)_2(3,5\text{-}DIPS)_4$ **after** irradiation interfered with *de novo* synthesis of other essential metalloelement-dependent enzymes required for repair of radiation injury and decreased survival. That is, only a fraction of the dose given **before** radiation remains when radiation-injury occurs and this fraction is useful in overcoming radiation-injury and increasing survival. Giving this large dose of $Cu(II)_2(3,5\text{-}DIPS)_4$ **after** irradiation may interfere with the synthesis of other metalloelement-dependent enzymes by enabling incorporation of the incorrect metalloelement, in this case Cu, when the larger dose is given **after** radiation injury. This possibility also offers a rationale for historical observations that large doses of Cu, Fe, Mn, and Zn compounds cause "radiosensitization", reducing survival and increasing radiation-induced mortality. Large doses of aminothiol radioprotectants that are effective in increasing survival of lethally irradiated mice when given **before** irradiation are also lethal when given **after** irradiation.

**Mechanistic Modes of Cu Chelate Action.**

The copper chelate of 5-diethylsulfonamoylsalicylic acid [Cu(II)(5-DESS)] has been recently reported to be effective in increasing survival of $LD_{50/30}$ (8 Gy, 1.4 to 1.3 Gy $min^{-1}$) irradiated mice. Treatment with 10, 20, 40, 60, 80, 100, or 120 μmol $kg^{-1}$ given sc **before** irradiation revealed that 80 or 120 μmol $kg^{-1}$ produced significant increases in survival, 68% ($P = 0.019$) or 92% ($P = 0.0008$), respectively, compared to vehicle-treated control mice [52].

$Copper(II)_2(3,5\text{-}DIPS)_4$ did not prevent hematopoietic and immunologic damage to irradiated mice, since 98-99% of myeloid progenitor cells were destroyed and immune reactivity was ablated. However, $Cu(II)_2(3,5\text{-}DIPS)_4$ did accelerate cellular repopulation of bone marrow and spleen following irradiation. Recovery of hematopoietic activity was accelerated whether mice were treated with $Cu(II)_2(3,5\text{-}DIPS)_4$ 3 hr **before** or 3 hr **after** irradiation and treatment increased levels of multipotential progenitor cells, which serve as a source of both myeloid and lymphoid progenitor cells, to account for recovered responses to T and B cell mitogens and antibody responses to a T-dependent antigen [see 48].

Radiorecovery and/or radioprotectant activity of Cu-containing compounds or complexes should be recognized as possibly facilitating activation of Cu-dependent proteins and enzymes required for repair of radiation injury, including induction of metallothioneine (MT) synthesis, and recovery of radiation-induced immuno-incompetency. In this context, cytokines such as interleukin-1 (IL-1), tumor necrosis factor, stem cell factor, and interleukin-12 have recently been suggested to restore function to tissues damaged by irradiation and protect mice from radiation lethality when given **before** irradiation [53]. In contrast, cytokines such as transforming growth factor β, interleukin-6, and interferon, given **before** irradiation, sensitize mice and increase radiation lethality [54]. It is most likely that there are complex cytokine interactions relevant to the amount of cytokine synthesized and timing of release in responding to the degree of radiation injury. Additional evidence for this comes from the correlation between survival of irradiated keratinocytes and the synthesis of IL-6 [55]. It is also known that changes in ceruloplasmin (Cp), a Cu-dependent multifunctional plasma enzyme, the plasma Zn containing $\alpha_2$-macroglobulin, and transferrin, the plasma Fe transporter, mediated responses to injury are regulated by IL-1 and other cytokines in facilitating tissue repair processes. Addition of inflammatory cytokines; IL-1α, IL-1β or inorganic Cu to hepatocyte culture medium increased mRNA for MT-1, MT-2, and Cp in a coordinated cytokine-activation of hepatic cells as an acute phase response to inflammation [56]. These observations offer a clearer understanding of the well-known cytokinemediated acute phase response to inflammation and the increase in Cp as an acute phase response to irradiation-induced inflammation.

Following the observed radioprotectant and radiorecovery activities of $Cu(II)_2(3,5\text{-}DIPS)_4$ as well as its facilitation of recovery of radiation-induced immuno-incompetency, the question still remained as to whether or not there was any correlation between these observations and repair or recovery of radiation-induced histopathology. To address this need histopathological studies of spleen, bone marrow, thymus, and small intestine were conducted in parallel with immunological studies of animals exposed to $LD_{50/30}$ irradiation (8.0 Gy, 1.55 Gy $min^{-1}$) alone, treatment with 40 μmol $kg^{-1}$ $Cu(II)_2(3,5\text{-}DIPS)_4$ alone, or irradiated and treated with a single dose of vehicle or $Cu(II)_2(3,5\text{-}DIPS)_4$. Treatment with $Cu(II)_2(3,5\text{-}DIPS)_4$ brought about repair of radiation-induced histopathology in these tissues [see 48]. Signs of recovery were demonstrable by the 7th to 14th day after irradiation. These observations lead to the general conclusion that although $Cu(II)_2(3,5\text{-}DIPS)_4$ does not prevent tissue injury when it is given **before** irradiation. Treatment with this chelate facilitates rapid tissue recovery from radiation-induced injuries. These recoveries from radiation-induced histopathology offer a biological marker for the re-establishment of the integrity of tissues known to be most vulnerable to irradiation.

The earlier review [see 48] also pointed out that $Cu(II)_2(3,5\text{-}DIPS)_4$ inhibits radiation-induced carcinogenesis and clastogenesis consistent with suppression of DNA replication and blockade of cell growth in the S phase as well as repair of DNA strand breaks. These effects are also consistent with reported anticlastogenic activity of $Cu(II)(glycinate)_2$, observed as a rapid recovery of bone marrow cell radiation injury and associated repair of DNA [see 48]. They are also consistent with the prevention or repair of DNA strand breaks by $Cu(II)_2(indomethacinate)_4$ in phorbol-treated human leukocytes and support Cu-dependent mechanisms for prevention or repair of radiation-induced DNA damage.

Whole body and local irradiation injury constitutes either systemic or local inflammation. Consistent with the antiinflammatory activities of Cu chelates [57], $Cu(II)_2(3,5\text{-}DIPS)_4$ has been found to be effective in facilitating repair of systemic inflammation caused by whole body irradiation (*vide supra*) and local inflammatory reactions. Ear swelling of female C3H mice treated 2 hr **before** porphyrin photosensitization and visible (480 to 645 nm) irradiation with 20, 40, or 80 μmol $kg^{-1}$ $Cu(II)_2(3,5\text{-}DIPS)_4$ produced dose-related decreases in porphyrin-mediated photosensitized ear swelling of 25%, 50%, or 90%,respectively, with a dramatic decrease in photosensitization-induced histopathology, suggested to be $O_2^{\cdot-}$-mediated inflammation due to activation of Xanthine Oxidase (XO) [58]. This inflammation was inhibited by Allopurinol, an inhibitor of XO, and Verapamil, a Ca-channel blocker (vide infra) suggested to prevent the isoenzyme transformation of Xanthine Dehydrogenase (XD) to XO [59], the form of this enzyme that synthesizes $H_2O_2$ and $O_2^{\cdot-}$. Consistent with this suggestion, ultraviolet epidermal cell injury of mouse ears (ultraviolet erythema, a recognized model of inflammation) was decreased with $Cu_2Zn_2SOD$ treatment and inhibition of $Cu_2Zn_2SOD$ by treatment with diethyldithiocarbamate exacerbated the "sunburn cell" formation but did not change ultraviolet radiation-induced ear swelling [60]. Ultraviolet irradiated human endothelial cells elaborate nitrogen monoxide (NO) which may account for vasodilation and ear swelling as an inflammatory response [61] following activation of inducible Nitrogen Monoxide Synthase (NOS).

The observed decrease in skin $Cu_2Zn_2SOD$ following a single ultraviolet irradiation was also reduced by pretreatment with liposomes containing $Cu_2Zn_2SOD$ [62]. Increased ultraviolet radiation-induced keratinocyte proliferation was also found to be associated with a decrease in total SOD activity which was due mainly to a decrease in $Cu_2Zn_2SOD$ [63]. Protection against ultraviolet radiation-induced chronic skin damage was also found with topically applied non-steroidal antiinflammatory agents [64]. These agents have been suggested to form Cu chelates *in vivo* [57], which may account for their inhibition or repair of ultraviolet radiation induced injury.

Copper(II)$_2$(3,5-DIPS)$_4$ was originally examined for radioprotectant activity based upon reports that $Cu_2Zn_2SOD$ had radioprotectant activity when given both **before** and **after** irradiation. However, subsequent reports suggested that superoxide disproportionation by $Cu_2Zn_2SOD$ did not account for its radioprotectant activity. Inactivated polyethylene-glycol derivatized $Cu_2Zn_2SOD$ was as effective as the enzymatically active derivative. The very real possibility that these proteins in extracellular spaces are in fact antigenic, although reduced antigenicity is always claimed, may lead to phagocyte elaborated IL-1 and/or some other cytokine(s) that initiates immune and repair responses that accounted for the observed increase in survival [see 48].

The hypothesis that there are radiolytic losses of essential metalloelement-dependent enzyme cofactors [see 48] has merit and is consistent with the report that there was a 20% loss of both $Cu_2Zn_2SOD$ and MnSOD in intestinal smooth muscle of rats immediately (1 hr) following irradiation (15 Gy). However, the activity of both enzymes increased over a 20 hr post-irradiation period to a level 45% higher than normal with a further increase in Mn SOD by 72 hrs post irradiation, a likely result of *de novo* synthesis in response to injury. It is noteworthy that $Cu(II)_2(3,5\text{-}DIPS)_4$, a SOD-mimetic, was subsequently found to be more effective as a radioprotectant and radiorecovery agent than $Cu_2Zn_2SOD$ and more effective than WR-2721 in reducing oxygen radical mediated colitis [65].

Adult CF male rats x-irradiated with 15 cGy $sec^{-1}$ $day^{-1}$ for 7 days followed by 7 days rest for either 3 months or 6 months showed duration related radiation-induced patchy skin hyalinization, which became completely hyalinized during the succeeding 6 months without irradiation [66]. The concentration of Zn in skin of these rats remained normal in both irradiated groups but by the end of 1 yr it had significantly ($P < 0.001$) decreased to 13 μg $gm^{-1}$ of tissue from 19 μg $gm^{-1}$. Skin Fe was elevated in both irradiated groups and the elevation increased from 100 μg $gm^{-1}$of tissue with 3-mo irradiation to 140 μg $gm^{-1}$ after 6-mo irradiation versus 68 μg $gm^{-1}$ for control skin. There was a further relative increase in skin Fe 1 yr after irradiation to 129 μg $gm^{-1}$ of tissue compared to 65 μg $gm^{-1}$ for control skin ($P < 0.001$). It was concluded that chronic exposure to low dose irradiation possibly causes a redistribution of Zn and Fe with concomitant hyalinization of collagen. The observed skin collagen pathology and altered essential metalloelement contents were interpreted as results of chronic inflammation due to chronic irradiation [66]. These changes in skin essential metalloelement contents are consistent with essential metalloelement-dependent enzyme responses required to overcome radiation injury.

Reduction in radiation-induced fibrosis with liposomal $Cu_2Zn_2SOD$ was studied using large white pigs γ-irradiated with a single dose of 160 Gy delivered to the skin. Treatment with 0.3 μmol $Cu_2Zn_2SOD$ applied 2 times per week for 3 wk caused shrinking of irradiation-induced fibrosis in all treated pigs. Scar regression was found to be 45% in length and width, 30% in depth, and 70% in area and volume. Histologic examination showed normal muscle and subcutaneous tissue surrounding the residual scar. This replacement of scar tissue by normal tissues and a 50% decrease in the linear dimensions of the scar were comparable to results obtained in a previous clinical trial of liposomal $Cu_2Zn_2SOD$ treatment [67].

Over expression of $Cu_2Zn_2SOD$ or MnSOD increased survival of 0.2 to 1.2 Gy (0.3 Gy $min^{-1}$) x-irradiated CHO cells and cells over-expressing MnSOD had greater survivals that those over-expressing $Cu_2Zn_2SOD$ [68]. As pointed out earlier [see 48] the SOD-mimetic reactivity of $Cu(II)_2(indomethacinate)_4$ was credited with the prevention of DNA strand-breaks in human leukocytes stimulated with phorbol-diester. This prevention or facilitated repair of DNA was observed over a concentration range of 2 to 9 μM $Cu(II)_2(indomethacinate)_4$.

Another possible mechanism offered to account for the radioprotectant and radiorecovery activities of Cu chelates involves Cu-dependent nuclear metabolism. It is known that 20% of cellular Cu is found in the nucleus. Copper has the highest bonding affinity for nucleic acids of all essential metalloelements. Nucleic acid phosphate and base donor sites yield Cu chelates, which are much more stable than Cu chelates of most amino acids and near the stability of protein chelates. This stability has been used to support suggestions of intrastrand and interstrand DNA bridging, which was recognized early as having a role in stabilization of the Watson-Crick double helix [see 48]. Higher physiologic concentrations account for inhibition of ribonuclease syntheses: initiation, nucleotide condensations, and release of newly formed mRNA. However, the original observation that injection of $Cu(II)Cl_2$ into rats induced the synthesis of mRNA coding for the synthesis of MT has been explained as due to the formation of a Cu-dependent DNA bonding protein *ACE1* or *CUP2*, the protein products of *S. cerevisiae* (ACE1) and *E. coli* (CUP2) genes, which recognize upstream activation sequences required for active transcription of *CUP1*, a MT gene as pointed out earlier [see 48] and subsequently [69]. Chaperones such as hAtox1 are Cu bonding proteins suggested to have roles in mediating Cu homeostasis in many cell types as cytosolic Cu-transporters, enabling the utilization of Cu in *de novo* synthesis of Cu-dependent enzymes and proteins [70]. Since radiation injury and injection of Cu chelates induce MT synthesis and increased MT synthesis correlates with increased survival, Cu chaperones such as Atox1, which mediate the role of Cu in transcription of $Cu_2Zn_2SOD$ and the anti-apoptotic bcl-2 genes [71], may represent important nuclear chemical responses in overcoming radiation injury.

Nuclease-Mimetic reactivity of Cu chelates has been recognized as valuable footprinting reagents useful in revealing details of RNA polymerase bonding to a *lac* promoter [see 48]. Predictable chromatin DNA cleavage by a chelate due to preferential bonding of the chelate to DNA spacer segments is also a useful characterizing feature. Interpretations that these and other DNA strand scissions resulting from Cu chelates are damaging [72,73] without recognizing that these scissions may be due to expected Cu chelate [74] or other essential metalloelement chelate [75] catalyzed diphosphate ester hydrolyses, which may be viewed as endonuclease- (or exonuclease-) mimetic reactivity, merit reconsideration. More recently, the antineoplastic action of the copper complex of Tambjamine was suggested to be due to DNA nuclease reactivity [76].

Like many Cu chelates, $Cu(II)_2(3,5\text{-}DIPS)_4$ has SOD-mimetic reactivity. While formation of ternary protein-$Cu(II)_2(3,5\text{-}DIPS)_4$ chelates formed with collagen or albumin have slightly less SOD-mimetic reactivity due to protein bonding to a free Cu bonding site [77], it is the formation of, as yet not unequivocally demonstrated, ternary albumin chelates formed *in vivo* [78,79] that were suggested to account for the observed rapid and pervasive tissue distribution of $^{67}Cu$ and $^{14}C$ labeled 3,5-DIPS within 0.5 hr following administration of $^{67}Cu(II)_2[3,5\text{-}DIP(Carboxy\text{-}^{14}C)S]_4$ [80].

Dietary supplementation with arginine, the substrate for NOS, required for the synthesis of NO has been found to accelerate intestinal mucosal regeneration and enhance bacterial clearance following radiation-induced rat enteritis [81]. Rats fed diet containing 4% arginine for 7 days following non-lethal local abdominal x-irradiation (11 Gy, 0.14 Gy $min^{-1}$) had significantly ($P<0.001$) increased mesenteric lymph node mass, decreased number of lymph node Gram-negative enteric bacilli and facultative anaerobic bacteria, CFU $gm^{-1}$ of lymph node, increased mucosal thickness, and jejunal and ileal villi height and number, as well as number of mucous cell villi compared to rats fed a diet containing 2% arginine or 4% glycine.

$Copper(II)_2(3,5\text{-}DIPS)_4$ has been suggested to facilitate NO mediated responses to inflammatory diseases [82,83]. However, it has also been found to decrease synthesis of NO by down-regulating nitric oxide synthase (NOS) enzyme activity which may account for the antiinflammatory activities of this and other Cu chelates as well as repair of radiation injury [82,83]. This is an especially interesting possibility since Cp, which increases in plasma following irradiation, has recently been reported to decrease NOS activity when incubated with endothelial cells. There was a time-dependent increase in cytosolic and particulate bonded copper, and this effect could be reproduced by incubation with $Cu(II)(histidinate)_2$ but not with $Cu(II)Cl_2$, which primarily bonded to all particulate fractions and less so to cytosolic components [84]. In addition, $Cu_2Zn_2SOD$ has recently been shown to release NO from S-nitrosothiols in the presence of glutathione [85] suggesting that $Cu_2Zn_2SOD$ might have a role in mediating constitutive or inducible NOS and facilitating NO-mediated physiological functions as well as mediating rat mucosal regeneration and bacterial clearance in radiation enteritis [81].

Recently a domain of Cp, distinct from the ferro-oxidase domain, has been reported to act as a glutathione-dependent peroxidase [86] and a glutathione-dependent alkyl hydroperoxidase [87], which offers an interesting accounting for a plasma Cu-dependent agent responsible for removal of lipid hydroperoxides. Ceruloplasmin has also been recently identified as a plasma Pituitary Adenylate Cyclase-Activating Polypeptide (PAC-AP) 1-38-bonding factor [88]. This factor is an alpha amidated peptide, one of many amide terminus neuronal hormone peptides synthesized by Cu-dependent $\alpha$-amidating mixed function oxidases [89]. PAC-AP has more recently been reported to prevent cytokine-induced pancreatic ß-cell cytotoxicity by inhibiting inducible NOS expression in ßTC cells [90]. The PACAP activity of Cp has also been reported to decrease cytokine-induced cytotoxicity by inhibiting inducible NOS in pancreatic $\beta$ cells [91]. These observations offer an accounting for the radioprotectant and radiorecovery activities of $Cu(II)_2(3,5\text{-}DIPS)_4$, since $Cu(II)_2(3,5\text{-}DIPS)_4$ has been reported to down-regulate, but not inhibit enzyme activity, NOS and decrease the synthesis of NO [81,82]. Alternatively, this Cu chelate as well as others may form ternary nitrogen monoxide chelates ($ON\text{-}CuL_2$) that have a role facilitating NO dependent processes [81,82].

The possibility remains that reported therapeutic actions of Cu chelates may be due to only a small portion of the administered dose bonded to serum albumin and transported to the site of action. That is, an effective antiinflammatory dose of $Cu(II)_2(3,5\text{-}DIPS)_4$ is 1 µmol per kilogram of body mass. This represents $10^{19}$ molecules per 70 kg adult treated with a supposed dose of 70 µmol. If only 0.01% of this dose was distributed throughout the body due to losses including excretion, each of the estimated $10^{12}$ cells in the adult body could experience contact with more than 1,000 molecules of this chelate. Since there is always an inflammatory-response mediated vasodilation at the site of disease-affected cells and greater perfusion of these affected cells by plasma filtrate, the concentration of drug in the environment of disease-affected cells could actually be greater than 1,000 molecules per cell. Smaller daily doses ranging down to 0.7 µmol per adult, which could be a more likely dose, would provide 10 molecules per cell with the loss of 99.99% of the administered dose. Such losses may be typical for all administered drugs.

**Radiation Protection and Recovery with Iron Chelates.**

Since radiolytic loss of Fe from Fe-dependent enzymes is also a possible consequence of ionizing radiation, non-toxic doses of $Fe(III)\text{-}(3,5\text{-}DIPS)_3$ were examined for radioprotectant activity. Doses ranging from 35 to 280 µmol $kg^{-1}$ given sc **before** irradiation produced increases of 50% to 84% survival of $LD_{50/30}$

irradiated (8 Gy, 1.55 Gy $min^{-1}$) male mice compared to vehicle-treated mice [see 48]. Treatment sc with 35 μmol Fe(III)-$(3,5\text{-DIPS})_3$ $kg^{-1}$ **before** $LD_{100/30}$ irradiation increased survival 300% compared to vehicle-treated mice (8% survival). Treatment with sc doses of 17.5, 35, or 70 μmol Fe(III)-$(3,5\text{-DIPS})_3$ $kg^{-1}$ **after** $LD_{100/30}$ irradiation failed to increase survival above the 8% observed for vehicle-treated control mice [91]. Radiorecovery of mice treated with this chelate and irradiated with smaller doses of irradiation remain to be determined.

Gamma irradiated (7 Gy, 2.9 Gy $min^{-1}$) young adult male DBA/2 mice treated with 16 μmol $kg^{-1}$ ip Fe(III) hematoporphyrin chloride (FeHp) either 1 and 4 days or 1, 4, and 7 days **after** irradiation had significantly ($P < 0.025$) increased spleen colony forming units (CFU-GM) by day 14 following irradiation [92]. Mice treated on days 1 and 4 **after** irradiation had significantly ($P < 0.025$) increased numbers of spleen cells by day 17 post-irradiation. IronHp treatment on days 1, 4, and 7 also caused a significant ($P < 0.005$) reduction in bone marrow cell number by day 14 and other post-irradiation periods of measurement. There was no increase in bone marrow CFU-GM observed for any treatment. Mice treated 24 hr **before** irradiation and 48 hr or 48 and 96 hr **after** irradiation produced significant ($P < 0.001$) increases in total spleen cell number and spleen CFU-GM by day 15 post-irradiation. The single treatment **after** irradiation caused a 5-fold larger increase in spleen CFU-GM number than non-irradiated or irradiated control values measured on day 15. The only significant ($P < 0.01$) change in bone marrow cellularity was a decrease in cell number for mice treated 4 hrs **before** and 48 and 96 hrs **after** irradiation. There were no significant changes in bone marrow CFU-GM number at any time of measurement on 11, 14, and 17 days post-irradiation, relative to irradiated control mice. These data were interpreted as due to hematostimulatory activity of FeHp with stimulation occurring following radiation-induced immunosuppression. Enhanced hematopoietic recovery was observed for spleen but not bone marrow of irradiated mice treated with FeHp. Treatment may have also increased the mobilization of progenitor cells from bone marrow to the spleen in addition to the FeHp stimulatory effects on spleen CFU-GM. Interestingly, the FeHp-mediated spleen hematopoietic response was greatest in mice treated 24 hrs **before** irradiation. There was no "protection" but resting hematopoietic stem cells may have been induced to begin the cell growth cycle and division, while actively dividing cells would have been at greater risk to radiation injury and cell death.

**Radiation Protection and Recovery with Manganese Chelates.**

The earlier review [see 48] cited a number of reports that inorganic Mn(II)$Cl_2$ given sc in the radioprotectant paradigm **before** irradiation increased survival and enhanced survival when co-administered with aminothiol radioprotectants. Following these reports Mn(II)$Cl_2$ was shown to repair radiation-induced skin and intestinal inflammation in mice [93]. Treatment with 180 μmol Mn(II)$Cl_2$ $kg^{-1}$ given ip to male C3H mice 24 hr **before** a single 20 to 52 Gy (5 Gy $min^{-1}$) local paw x-irradiation or 12 to 21 Gy abdominal irradiation fed to repair of acute intestinal and skin radiation-injury. Skin and intestinal MT was not increased by administration of $MnCl_2$ although a six-fold increase was found in liver. These authors failed to point out that the increase in liver MT was associated with the increase in survival of mice treated with Mn(II)$Cl_2$.

Based upon the radioprotectant and radiorecovery activities observed for non-toxic doses of Mn-$(3,5\text{-DIPS})_2$, which may either be a spherically symmetric trinuclear chelate, $Mn(III)_2Mn(II)\mu_3O(3,5\text{-DIPS})_6$, or a linear trinuclear chelate, $Mn(II)_3(3,5\text{-DIPS})_6$, was examined for radioprotectant and subsequently for radiorecovery activity [see 48]. Subcutaneous treatment with 90, 120, or 150 μmol Mn-$(3,5\text{-DIPS})_2$ $kg^{-1}$ 3 hr **after** a $LD_{70/30}$ irradiation again demonstrated that all three doses were effective, producing 200%, 229% or 214% increases in survival above vehicle-treated mice (28% survival) [93].

Treatment with 150 μmol Mn-$(3,5\text{-DIPS})_2$ $kg^{-1}$ **before** $LD_{100/30}$ irradiation and then 30, 60, or 120 μmol $kg^{-1}$ on three successive days **after** irradiation produced survivals of 28%, 12%, or 16% respectively versus 0% survival for vehicle-treated mice [94]. Treatment with 90, 120, or 150 μmol $kg^{-1}$ 3 hrs **after** $LD_{70/30}$ irradiation increased survival from 7% for the vehicle-treated mice to 21%, 23%, or 22%, respectively, 200% ($P = 0.00007$), 229% ($P = 0.000005$), or 214% ($P = 0.00002$) increases in survival compared to vehicle-treated mice [94]. Treatment with 150 μmol $kg^{-1}$ 3 hr **before** $LD_{100/30}$ irradiation (10 Gy, 1.4 Gy $min^{-1}$) and 90, 180, or 360 μmol $kg^{-1}$ on day 2, 4, and 6 **after** irradiation produced 28% ($P = 0.007$), 12%, or 16% survival, respectively, versus 0% survival for vehicle-treated mice. Treatment with 120, 240, or 480 μmol Mn-$(3,5\text{-DIPS})_2$ $kg^{-1}$ **before** $LD_{100/30}$ irradiation revealed that only the 240 and 480 μmol $kg^{-1}$ doses were effective, producing survivals of 16% or 27%, respectively, with no surviving control mice [94]. Treatment with doses of 60, 120, or 240 μmol Mn-$(3,5\text{-DIPS})_2$ $kg^{-1}$ **after** $LD_{100/30}$ irradiation revealed that only the 120 μmol $kg^{-1}$ dose was effective, increasing survival 233% above control mice (12% survival) [94].

Oral treatment with Mn-(3,5-DIPS)$_2$ **before** or **after** $LD_{100/30}$ irradiation increased survival and recovery of radiation-induced loss of body mass [94]. Treatment with 120, 240, or 480 μmol Mn-(3,5-DIPS)$_2$ $kg^{-1}$ orally 3 hr **before** irradiation or 60, 120, or 240 μmol $kg^{-1}$ 3 hr **after** irradiation increased survival and allowed greater recovery of radiation-induced loss of body mass compared to vehicle-treated mice. Treatment with 120, 240, or 480 μmol Mn-(3,5-DIPS)$_2$ $kg^{-1}$ orally 3 hr **before** irradiation produced 4%, 16%, or 27% survival versus 0% for vehicle-treated mice. Treatment with 20, 120, or 240 μmol Mn-(3,5-DIPS)$_2$ $kg^{-1}$ 3 hr **after** irradiation produced 12%, 40%, or 4% survival versus 12% survival for vehicle-treated mice.

**Mechanistic Modes of Mn Chelate Action.**

A possible Mn-(3,5-DIPS)$_2$ mechanism of action in overcoming inflammation due to radiation injury is the down-regulation of NOS when excess NO is produced as part of the inflammatory response [82]. Alternatively, activation of Mn-dependent guanylyl cyclase [95], the receptor for NO, by Mn-(3,5-DIPS)$_2$ may serve to restore normal physiologic functions involving NO-mediated processes. This understanding is consistent with the suggestion that a ternary NO-Mn-(3,5-DIPS)$_2$ complex might form *in vivo* [82] and have roles in mediating NO function [83].

The Mn-Desferrioxamine (MnDFO) SOD-mimetic chelate has also been found to be effective in protecting against riboflavin-mediated ultraviolet (365 nm, 2 to 9 J $cm^{-2}$) phototoxicity [96]. Photohemolysis of erythrocytes was decreased with 500 μM MnDFO. Treatment with 50 or 100 μM MnDFO did not change $^3$H-Thymidine incorporation in irradiated (5 J $cm^{-2}$) GH-U1 human urinary bladder carcinoma cells or irradiated (10 J $cm^{-2}$) phytohemaglutin-stimulated lymphocytes compared to radiation alone. Higher concentrations of MnDFO were toxic to these cells.

Hahn et al. [97] and Darr et al. [98,99] suggested that MnDFO is superior to Cu chelates as an SOD-mimetic because Cu chelates dissociate in the presence of protein. This suggestion was offered even though Cu chelates are much more effective in disproportionating $O_2^-$ at an appropriate physiological pH and more effectively treat many disease states (see 2). They overlook the fact that Mn chelates also dissociate in the presence of protein or other biochemically important macromolecules *via* ligand exchange. However, these losses may not prevent their use in treating diseases (*vide supra et infra*). More importantly they overlook the possibility that the presence of protein such as albumin can lead to ternary Cu or Mn chelate formation with enhanced stability and serve as Cu or Mn chelate transporting agents in vivo [77-79]. Consequently, the view that Cu or Mn chelates can not be effective SOD-mimetics in preventing $O_2^-$-mediated pathology, despite many observations to the contrary is seriously flawed. Finally, Mn chelates will not serve to facilitate the activation of Cu-dependent enzymes nor will Cu chelates facilitate activation of Mn-dependent enzymes.

The SOD-mimetic reactivity of Mn(III)tetrakis-1-methyl-4-pyridylporphyrin pentachloride [Mn(III)TMPyP] was recently suggested to account for the restoration of NO-mediated neurotransmission and serves as a lead compound in the development of SOD-mimetics as therapeutic agents for the treatment of neuropathies associated with oxidant stress [100].

To examine the possibility that treatment with a combination of essential metalloelement chelates might be effective in increasing survival of lethally irradiated mice, Cu(II)$_2$(3,5-DIPS)$_4$ and Mn-(3,5-DIPS)$_2$ were studied using a factorial design [101]. Treatment with 10, 20, or 40 μmol Cu(II)$_2$(3,5-DIPS)$_4$ $kg^{-1}$ **before** $LD_{90/30}$ irradiation (9 Gy, 1.25 Gy $min^{-1}$) produced survivals of 28%, 28%, or 36% survival, respectively, 133%, 133%, or 200% increases above vehicle-treated mice (12% survival). Increasing the dose of Cu(II)$_2$(3,5-DIPS)$_4$ increased survival in these more rigorously radiation-injury-challenged mice. Treatment with 10, 20, or 40 μmol Mn-(3,5-DIPS)$_2$ $kg^{-1}$ **before** $LD_{90/30}$ irradiation (9 Gy, 1.25 Gy $min^{-1}$) produced survivals of 36%, 20%, or 24% survival respectively, 200%, 67%, or 100% increases in survival respectively, compared to the same vehicle-treated mice. Increasing the dose of Mn-(3,5-DIPS)$_2$ did not produce a dose-related increase in survival. Using combinations of 10, 20, or 40 μmol Cu(II)$_2$(3,5-DIPS)$_4$ and 10, 20, or 40 μmol Mn-(3,5-DIPS)$_2$ $kg^{-1}$ revealed that the combination of 20 μmol Cu(II)$_2$(3,5-DIPS)$_4$ plus 20 μmol Mn-(3,5-DIPS)$_2$ $kg^{-1}$ produced 48% survival, a 300% ($P = 0.01$) increase above the same vehicle-treated mice. While increasing the dose of Cu(II)$_2$(3,5-DIPS)$_4$ from 10 to 40 μmol $kg^{-1}$ increased survival, increasing the dose of Mn-(3,5-DIPS)$_2$ from 10 to 40 μmol $kg^{-1}$ did not increase survival and increasing doses of Mn-(3,5-DIPS)$_2$ beyond 20 μmol $kg^{-1}$ given in combination with Cu(II)$_2$(3,5-DIPS)$_4$ were associated with decreases in survival or no increase in survival due to combination-treatment. This apparent negative effect on survival associated with increasing doses of Mn-(3,5-DIPS)$_2$ given in combination with Cu(II)$_2$(3,5-DIPS)$_4$ may be due to an interference of Mn in *de novo* synthesis of Cu or some other metalloelement-dependent enzymes wherein the incorporation of Mn leads to a reduction in activity of non-Mn metalloelement-dependent enzymes requiring a metalloelement other than Mn for activation.

Irradiation-Induced esophagitis, micro-ulceration, and esophageal stricture as well as cytokine mRNA elevations and squamous cell apoptosis were dramatically decreased in male C3H/HeNsd mice with intra-esophageal instillation of mouse MnSOD-plasmid/liposome (SOD2-PL) 24 hr **before** 35 or 37 Gy local esophageal x-irradiation (58). Mice bearing orthostatic thoracic tumors treated with mouse SOD2-PL also evidenced esophageal transgenic SOD2 mRNA [102]. These results were suggested to provide support for human SOD2-PL gene therapy in a fractional irradiation paradigm to avoid acute and chronic radiation-induced side effects in a human clinical trial of pulmonary tumor irradiation [102]. Increased survival was also observed at both levels of irradiation. A significant increase in surviving fraction (1.0, P = 0.0291) versus 0.5 for control liposome treated mice was observed for 35 Gy irradiated mice treated with 100 μg SOD2-PL/mouse given by the intra-esophageal route of administration with a significant (P = 0.015) decrease in esophagitis in this 30-day study. Mice irradiated with 37 Gy and treated with 200 μg SOD2-PL/mouse or 10 μg SOD2-PL/mouse by intra-esophageal instillation experienced increased surviving fractions of 0.5 (P = 0.05) or 0.7 (P = 0.0119) respectively compared to non-treated irradiated mice in this 60-day study [102]. While intra-trachael instillation of transgene **before** irradiation protected C57BL/6J mice from whole-lung irradiation-induced alveolitis/fibrosis, there was no significant lung protection in SOD2-PL treated SP1-SOD2 or FeVB/NHsd transgenic mice. Over expression of either human SOD2 or murine Sod2 in the lungs of these transgenic mice did not provide local 11 to 15 Gy irradiated whole-lung or alveolar type II cell protection [103]. Over expression of SOD2 in SP-1 SOD2 mice may have made these mice more sensitive to radiation injury. However, these studies may have been seriously flawed since mice developing pulmonary distress were killed, preventing the possible observation that treated mice might have overcome this radiation-induced inflammatory complication.

**Radiation Protection and Recovery with Zinc Chelates.**

As pointed out in our earlier review [see 48] treatment with Zn compounds dramatically increased survival of lethally irradiated mice. In the interim it was shown that fractional whole-body γ-irradiation of male BALB/C mice given a total doses of 0.5, 1, or 1.5 Gy induced an increase in DNA-protein cross-links in the thymus, spleen, brain, and liver. However, when these mice were given drinking water containing 154 μM $Zn(II)SO_4$ for 30 days DNA-protein cross-links were dramatically decreased to levels observed in all of these tissues from non-irradiated mice [104].

Fibroblasts cultured in medium containing 100 μM $Zn(II)Cl_2$ had repaired ultraviolet radiation-induced DNA strand-breaks and delayed in apoptotic loss of nucleosomes [105] Cells cultured with 100 μM $Zn(II)Cl_2$ and irradiated with 1.4, 2.9, 3.6, or 5 $J/cm^2$ actually evidenced more double-stranded DNA than non-irradiated cells. This result suggests that $Zn(II)Cl_2$ prevents and/or repairs DNA strand-breaks Similarly, apoptosis was always less in $Zn(II)Cl_2$ supplemented cells at all times of measurement; 0, 3, 6, 8, 11, 12, and 18 hrs after irradiation consistent with prevention and/or repair of irradiation-induced injury leading to apoptosis. Gamma irradiation-induced thymocyte DNA strand-breaks and apoptosis were synergistically decreased with combinations of $Zn(II)SO_4$ and diethyldithiocarbamate, a compound known to form $Zn(II)(diethyldithiocarbamate)_2$ [106].

Since addition of Zn (0.15 μM) to culture medium **did not protect human melanoma cells** through a radiation dose range of 1.5 to 8.0 Gy (0.85 Gy $min^{-1}$). It had been suggested earlier [see 48] that the protective effect of Zn was due to the facilitation of some homeostatic mechanism which was suggested to be induction of MT synthesis [see 48]. Studies of MT gene regulation identified the involvement of bacterial lipopolysaccharide, IL-1, interferon, and other immunostimulants with control of MT synthesis in the liver. These observations coupled with the observation that IL-1 has radioprotectant activity [54] support the hypothesis that MT synthesis has a role in reducing radiosensitivity. Induction of MT synthesis is a physiological response to oxidant stress as described for IL-1 or endotoxin-mediated responses to this stress as well as other cytokine-mediated responses to radiation injury [58,59].

As pointed out earlier [see 48] $Zn(II)(aspartate)_2$ is known to have radioprotectant activity. An important question as to whether or not this compound protected neoplastic cells from radiation injury was answered when ip treatment with 90 μmol $kg^{-1}$ $Zn(II)(aspartate)_2$ did not inhibit γ-ray radiotherapeutic reduction of tumor volume using three adenocarcinomas of the colon or Ewings sarcoma implanted immunosuppressed mice [107]. $Zinc(II)(aspartate)_2$ also significantly reduced radiation-induced decrease in hematocrit and thrombocyte, erythrocyte, and leukocyte numbers, an indication of spleen and/or bone marrow precursor cell sparing effect or rapid recovery of immunocompetence. $Zn(II)(aspartate)_2$ permitted a greater reduction in tumor volume than cysteamine [mercaptoethylamine (MEA)], AET, and WR-2721 and it was as effective as a syngeneic bone marrow transplant [107].

Doses of 90 μmol Zn(II)(aspartate)$_2$, 80 μmol Zn(II)(histidinate)$_2$, 80 μmol Zn(II)(orotate)$_2$, or 122 μmol Zn(II)(acetate)$_2$ kg$^{-1}$ given ip 3 hr **before** irradiation (3.5 to 7.25 Gy) facilitated post-irradiation recovery of hematocrit, thrombocytes, erythrocytes, and leukocytes in C3H male and female mice [108]. Generally, Zn(II)(aspartate)$_2$, was more effective than the other Zn chelates and administration along with WR-2721 produced a marked synergistic increase in recovery of hematocrit and thrombocytes. Zinc(II)(aspartate)$_2$ was also the only Zn chelate that did not inhibit radiation-induced reduction in growth of adenocarcinoma-implanted and immunosuppressed mice [108].

Small non-toxic doses of Zn(II)(aspartate)$_2$, 45 μmol kg$^{-1}$ ip, and WR-2721 , 175 μmol kg$^{-1}$ ip, given to female C3H mice 30 min or 10 min **before** $LD_{100/30}$ γ-irradiation (10.5 Gy, 0.2 Gy min$^{-1}$) produced a synergistic increase in survival of 83% versus 0% for vehicle-treated control mice [109]. Zinc(II)(aspartate)$_2$, 45 μmol kg$^{-1}$ ip, and WR-2721, 1,400 μmol kg$^{-1}$ ip, given to female C57BL/6 mice 30 min or 10 min **before** a fractionated $LD_{100/30}$ γ-irradiation (5 x 1.9 Gy) also produced a synergistic increase in survival of 83% versus 0% for vehicle-treated control mice. Fractionated whole body radiation-induced carcinogenesis leading to lymphoid tumors was dramatically decreased from 90% to 9% in C57BL/6 mice treated with Zn(II)(aspartate)$_2$, 45 μmol kg$^{-1}$ ip, and WR-2721, 1,400 μmol kg$^{-1}$ given ip. Thus this combination treatment dramatically decreased radiation-induced lymphoid tumors [108].

The earlier review [see 48] pointed out that treatment with 60 μmol Zn(II)$_2$(3,5-DIPS)$_4$ kg$^{-1}$ produced 95% survival in $LD_{50/30}$ (8 Gy, 1.55 Gy min$^{-1}$) irradiated male mice. Acute toxicity of this chelate was also due to CNS depression with a sedative-hypnotic dose of 123 μmol kg$^{-1}$. However, the effective dose of Zn(II)$_2$(3,5-DIPS)$_4$ was only 1/6 of the acute toxic dose 381±75 μmol kg$^{-1}$.

**Mechanistic Modes of Zn Chelate Action.**

Since Zn chelates have no SOD-mimetic reactivity, activation or synthesis of $Cu_2Zn_2$SOD, MT, or other Zn-dependent enzymes including endonucleases and other known and suspected Zn-dependent transcription and translation processes [110] must at least partially account for their radioprotectant or radiorecovery activity. Another partial accounting may be attributed to protection of spleen and bone marrow stem cells by decreasing DNA replication and blockade in the S growth phase as observed for CHO cells treated with Zn(II)$Cl_2$ as pointed out earlier [see 48].

A 100 μmol kg$^{-1}$ dose of Zn(II)-Desferrioxamine [Zn-DFO] produced 60% survival in whole body $LD_{50/30}$ γ-irradiated female C3H mice treated 0.5 hr **before** irradiation while Desferioxamine had no radioprotectant activity [111]. There was no radioprotective effect observed in studies of cultured V79 Chinese hamster cells. However, Zn-DFO concentration dependently prevented radiation-induced plasmid DNA strand-breaks through a concentration range of 0.5 to 2 mM.

Feeding an "antioxidant" diet containing 55 μmol Zn(II)(gluconate)$_2$ kg$^{-1}$ day$^{-1}$ to C57BL male mice for 45 days **before** irradiation(6 Gy, 1.8 Gy min$^{-1}$) caused an inhibition of TRPM-2, a marker gene for apoptosis expression both **before** and after irradiation [112]. The bcl-2 mRNA, which is involved in the prevention of apoptosis, and splenocyte number increased steadily after irradiation. The expression of sod-I gene increased and remained elevated for 24 hr, the last time of measurement after irradiation, and synthesis of the cat (catalase) gene increased slightly shortly after irradiation and decreased by 3 hr after irradiation. Preliminary studies under the same experimental conditions revealed a decrease in frequency of radiation-induced mutations as evidenced by changes in the hypoxanthine-quanine phosphoribosyl-transferase locus of splenocytes from mice fed the antioxidant diet containing Zn(II)(gluconate)$_2$. The maximum antimutagenic effect was detected by the 6$^{th}$ week of diet administration.

**Radioprotectant and Radiorecovery Activity of Calcium-Channel Blockers.**

New evidence provided by Floersheim and his colleagues document radioprotectant and radiorecovery activities for some Ca-channel blockers [113-116]. These reports are consistent with the earlier suggestion of Athar et al. [59] that Verapamil, a Ca-channel blocker, prevents radiation injury-induced conversion of Xanthine Dehydrogenase to Xanthine Oxidase, which synthesizes $O_2^-$.

Diltiazem, a benzothiadiazepine Ca-channel blocker, given sc as the hydrochloride salt in distilled water to female mice at doses of 61, 122, or 244 μmol kg$^{-1}$ 15 min **before** $LD_{100/30}$ (10.5 Gy, 0.15 Gy min$^{-1}$) irradiation produced 17%, 58%, or 93% survival [113]. A dose of 244 μmol kg$^{-1}$ given sc 10 min **after** irradiation produced 42% survival for this radiorecovery paradigm. Nifedipine, a dihydropyridine Ca-channel blocker, given ip at a dose of 4 μmol kg$^{-1}$ in an ethanol vehicle, which provided 50 mmol ethanol kg$^{-1}$, 30 min

**before** $LD_{100/30}$ irradiation (8.5 Gy, 0.9 Gy $min^{-1}$) produced 100% survival and the vehicle produced 61% survival, a difference of 39% [113]. Treatment with 5 μmol Nimodipine $kg^{-1}$, another dihydropyridine Ca-channel blocker, produced 82% survival whereas the vehicle containing 43 mmol ethanol produced 42% survival. Distilled water-treated mice experienced 0% survival. Male mice treated ip with 10 μmol Nimodipine $kg^{-1}$ 30 min **before** $LD_{100/30}$ 8.1 Gy (0.9 Gy $min^{-1}$) irradiation produced 75% survival while treatment with 9 μmol $kg^{-1}$ ip Nifedipine (a dihydropyridine) produced 67% survival and the ethanol vehicle produced 30% survival. Injection of distilled water produced 0% survival. However, ip treatment with a dose of 10 μmol $kg^{-1}$ Nimodipine or 9 μmol $kg^{-1}$ Nifedipine given **before** irradiation produced 58% survival while the group treated with the ethanol vehicle had 0% survival [113].

Isradipine, another dihydropyridine Ca-channel blocker, given at a dose of 74 μmol $kg^{-1}$ ip **before** irradiation (9 Gy) produced 58% survival. Treatment with 278 μmol $kg^{-1}$ Nitrendipine (a dihydropyridine Ca-channel blocker), ip, produced 29% survival. Verapamil, a substituted aminopropylnitrile Ca-channel blocker, given at a dose of 35 μmol $kg^{-1}$ ip and Flunarizine, a piperazine Ca-channel blocker, given **before** irradiation (9 Gy) at a dose of 62 μmol $kg^{-1}$ orally did not produce any increase in survival compared to the vehicle-treated group [113]. Based upon the efficacy of Verapamil in facilitating repair of photosensitization-radiation injury and its prevention of the XD to XO conversion as reported by Athar et al. [58,59], this mechanistic feature of Ca-channel blocker activity must be taken into account in examining their potential for radioprotectant activity. It would also be useful to examine this mechanistic aspect of all radiorecovery agents.

Floersheim suggested that Ca-channel blockers might also act by blockade of cell death due to Ca influx into cells following radiation injury [113]. However the antioxidative effects of Ca-channel blockers *via* scavenging of free radicals was suggested as an alternative mechanism of action, which could involve stable essential metalloelement chelate formation following hydrolysis of ester functional groups *in vivo*. Radical scavenging reactivity of aminothiol radioprotectants and formation of their disulfides was also suggested as a mechanism of their action in overcoming radical-mediated radiation injury [113]. A chelate mechanism for radical scavenging by aminothiols is also plausible. Variations in activity of Ca-channel blockers were suggested to be due to variations in bioavailability and receptor-bonding interaction [113].

Benzothiadiazepines and dihydropyridine Ca-channel blockers may also serve as chelating agents and thus facilitate delivery of essential metalloelements to cells for *de novo* synthesis of essential metalloelement-dependent repair enzymes. Calcium-Channel blockers have also been reported to decrease radiation-induced esophagitis as a consequence of thoracic irradiation. Variation in blood flow was proposed to explain the modified x-ray sensitivity of mouse tumor [113]. Calcium-Channel blocker-induced hypotension and tissue anoxia was negated as the cause of radioprotectant activity of these agents, since other agents causing these conditions had no radioprotecting activity [113]. Subsequently Floersheim reported [114] that 9 μmol Nifedipine $kg^{-1}$ given ip 3 hr **before** $LD_{100}$ irradiation (9.8 Gy, 0.9 Gy $min^{-1}$) produced 83% survival. Again, even though the radiation dose was a $LD_{100/30}$ dose, the 96% ethanol vehicle produced 61% survival while an 18% ethanol vehicle produced 28% survival.

A dose of 244 μmol Diltiazem hydrochloride $kg^{-1}$ given sc to $LD_{100/30}$ (10 Gy, 0.15 Gy $min^{-1}$) irradiated male C3H mice produced 90% survival ($P < 0.005$) with treatment 30 min **before** irradiation or 75% survival when given 10 min **before** irradiation. Treatment with 122 μmol $kg^{-1}$ under these conditions and 30 min **before** irradiation produced 42% survival ($P < 0.01$) compared to 8% survival for vehicle (distilled water)-treated mice. When given ip at 244 μmol $kg^{-1}$ 10 min **before** irradiation there was 100% survival ($P < 0.005$) but only 80% survival ($P < 0.005$) when given 30 minutes **before** irradiation compared to 17% survival for vehicle (distilled water)-treated mice. Treatment of female C3H mice given a 10.5 Gy dose of irradiation and a dose of 244 μmol $kg^{-1}$ Diltiazem ip 10 min **before** irradiation produced 54% survival ($P < 0.005$) compared with 2% survival for vehicle (distilled water)-treated mice (70). When these mice were treated 30 min **before** irradiation 59% survived ($P < 0.005$). Treatment 120 min **before** irradiation produced only 19% survival ($P < 0.025$).

Diltiazem given in distilled water to $LD_{100/30}$ (10 Gy, 15 cGy $min^{-1}$) irradiated female BALB/c mice **before** treatment with 244 μmol $kg^{-1}$ ip or sc produced 100% survival ($P < 0.005$) compared to vehicle-treated mice. The same 244 μmol $kg^{-1}$ dose given sc or ip to C57BL6, NMRI, or MAG mice produced significant ($P \leq 0.01$) strain-related differences in efficacy: 100%, 67%, or 8% survival respectively [114].

Nifedipine given 30 min **before** irradiation at doses of 2.3, 4.5, or 9 μmol $kg^{-1}$ ip to $LD_{100/30}$ (10.5 Gy, 0.15 Gy $min^{-1}$) irradiated female C3H mice produced 0%, 40%, or 79% ($P \leq 0.05$) survival, respectively, compared to 6% survival for ethanol vehicle-treated control mice [114]. Nitrendipine given at doses of 139 or 278 μmol $kg^{-1}$ ip in distilled water to these mice 30 min **before** irradiation produced significant ($P < 0.005$) increases of 25% or 29% survival respectively. When they were given 120 min **before** irradiation they produced 22% or 30% survival ($P \leq 0.01$) versus 0% survival for distilled water-treated control mice [114]. Verapamil, given to these mice at doses of 35 μmol $kg^{-1}$ ip or Flunarizine, a piperazine Ca-channel blocker, given to these mice at doses of 62 μmol $kg^{-1}$ ip at 30 or 120 min **after** irradiation did not produce any increase in survival compared to the 0% survival for vehicle-treated mice [114].

Combinations of 61, 91, 122, or 244 μmol $kg^{-1}$ Diltiazem and 7.5, 15, 30, 45, or 91 μmol $kg^{-1}$ Zn(II)$(aspartate)_2$ given to female C3H mice **before** irradiation were studied in a factorial designed experiment. Survivals for Diltiazem-treated mice were 33%, 44%, or 91% respectively while only the 91 μmol $kg^{-1}$ dose of Zn(II)$(aspartate)_2$ was effective and produced 92% survival in these $LD_{100/30}$ (10.5 Gy, 0.15 Gy $min^{-1}$) irradiated mice. However survival increased for nearly all combinations of Diltiazem and 45 μmol $kg^{-1}$ Zn(II)$(aspartate)_2$ to 50%, 100%, or 92% [114].

Combinations of Diltiazem and Nifedipine were also studied using the factorial paradigm. A synergistic increase in survival was found for increasing active doses of Nifedipine and Diltiazem from 61 to 122 μmol $kg^{-1}$ when given **before** irradiation [114]. No synergism was found for combinations of active doses of Diltiazem, Nitrendipine, or Nifedipine and WR-2721. Synergism was observed for combinations of Diltiazem ranging from 7.5, 15, 30, 61, or 122 μmol $kg^{-1}$ and 17.5, 26, or 35 mmol DMSO $kg^{-1}$, reaching 100% survival for combinations of 122 μmol $kg^{-1}$ Diltiazem and 17.5, 26, or 35 mmol DMSO $kg^{-1}$ in $LD_{100/30}$ irradiated C3H mice treated **before** irradiation [114].

Koch [115] suggested that enhanced local blood flow caused by Ca-channel blockers might be responsible for increased radiosensitivity due to the oxygen effect. The exchange by Koch [115] and Floersheim [116] offered a number of speculations concerning Ca-channel blockade in radiosensitization and radioprotection. These related to dose, chemical class, a possible role for small molecular mass solvent molecules, as well as effects on metabolizing enzymes.

Interestingly, calcium was found to be elevated in heart, liver, lung, spleen, and kidney of adult male albino rats but decreased in these tissues of female rats two months after 5 Gy γ-irradiation [117]. While cardiac Fe was elevated, Fe content decreased in all other tissues of male rats. There were no changes in heart and lung Fe in female rats while liver, spleen, and kidney content decreased in female rats. Zinc was elevated in the heart, decreased in lung and spleen, and unchanged in liver and kidney of both male and female rats [117].

**Radioprotectant Activity of Serotonin and Acyl Melatonin Homologs.**

A review of Melatonin, N-acetyl-5-methoxytryptamine, antioxidant effects pointed out that Melatonin is relatively non-toxic and protects against ionizing radiation injury [118]. This review cited the report of Blickenstaff et al. [119] as having shown that Melatonin, the acetylated methoxyether of Serotonin, 5-hydroxytryptamine, produced 75 % survival in $LD_{100/30}$ 9.5 Gy whole-body irradiated male Swiss ND4 mice. This was an increase in radiation protection compared to Serotonin, which produced only 20 % survival in the same study. Intraperitoneal treatment of these mice with soybean oil solutions or suspensions of 1.1 mmol $kg^{-1}$ acetyl (Melatonin), propionyl, butanoyl, pentanoyl, hexanoyl, octanoyl, decanoyl, or hexadecanoyl analogs of 5-methyltryptamine 30 min **before** irradiation produced survivals of 0 %, 43 % ($P = 0.0045$), 32 %, 75 %, 95 %, 65 %, or 48 % respectively. In this experimental paradigm a 0.78 mmol WR-2721 $kg^{-1}$ treatment produced 50 % survival. Radioprotectant activities of all Melatonin analogs were significantly different from vehicle-treated mice and the activity of Melatonin was about equal to that of WR-2721. The increase in radioprotectant activity was greatest for the hexanoyl and octanoyl derivatives, showing an increase in activity with increasing lipophilicity. However, further increasing lipophilicity with the formation of still longer chain amides lead to a decrease in activity, as is usually the case when lipophilicity is increased beyond some optimal value. The observed radioprotectant potency as well as a lack of Melatonin toxicity was suggested to support further study of Melatonin and these acyl homologs in circumstances of human radiation injury [118].

These compounds can participate in coordinate-covalent bond formation with essential metalloelements. The considerable enolic character of the amide carbonyl group and the proximity of the weakly acidic indole amine offer possible coordination sites.

Vijayalaxmi et al. found that treatment 1 hr **before** $LD_{50/30}$ 8.15 Gy whole-body irradiation with ip doses of 0.55 or 1.1 mmol Melatonin $kg^{-1}$ suspended in soybean oil produced survivals of 50 % or 85 % ($P = 0.0080$) respectively in CD2 mice versus 50 % survival for vehicle-treated mice [120].

Karbownic et al. [121] recently reported that 440 μmol Melatonin $kg^{-1}$ given as a soybean oil suspension ip to male Sprague-Dawley rats four times at 120, 90, 60, and 30 min **before** whole-body 8 Gy irradiation completely prevented the formation of 8-hydroxy-2'-deoxyguanosine and microsomal membrane rigidity in hepatocytes. Both of these damages are consequences of ionizing irradiation due to DNA or protein and lipid damage. It is noteworthy that these repairs of radiation injury were observed as soon as 12 hr after treatment and irradiation.

**Radioprotectant Activity of Substituted Anilines.**

Using the same experimental paradigm that allowed the observation of radioprotectant activity for Melatonin homologs, Blickenstaff et al. [122] found that a series of substituted anilines had radioprotectant activity. Intraperitoneal treatment of male Swiss ND4 mice with soybean oil solutions or suspensions of 780 μmol $kg^{-1}$ 4-aminobenzophenone, 4-aminopropiophenone, or the ethyl ketal of 4-aminopropiophenone derivatives of aniline 30 min **before** $LD_{100/30}$ 9.5 Gy irradiation produced survivals of 100%, 100 % or 95 % respectively versus 4 % survival for vehicle-treated mice. This radioprotectant paradigm also revealed a range of 88 % to 55 % survivals for 780 μmol $kg^{-1}$ treatments using: 4-Br; 4-CN; 3-Br; 4-$NO_2$; 4-$COCH_3$; 4-Cl, 2-$CF_3$; 4-Cl, 3-$NO_2$; 4-$CO_2CH_3$; 3-Cl; 4- Cl; 4-$CF_3$; 4-$SCH_3$; 4-I; and 4-Cl, 3-$CF_3$ substituted anilines, survivals ranged from 40 % down to 5 % for 3,4-diCl; H (aniline); 4-$NH_2$; 4-$CH_3$; 4-$CONH_2$; 4-Cl, 2-$NO_2$; 4-Cl,2-$CO_2H$; 4-$COC_6H_4NH_2$; 4-$CH_2CH_2OH$; 4-$CONHNH_2$; 4-OH; 4-Cl,2-$OCH_3$,5-$CH_3$; and 4-F substituted anilines, and 0 % survival for 2-Cl; 2-Br; 3,4,5-triCl; and 2-$COC_6H_5$,4-Cl substituted anilines [122].

In addition, Blickenstaff et al. [122] reported 95 % survival for 780 μmol $kg^{-1}$ 2-$NH_2$,5-Cl-pyriding and 2-Cl,5-$NH_2$-pyridine treated mice, 79 % survival for 2-$NH_2$,5-Br-pyridine treated mice; 5 % survival for 2-$NH_2$,4-$CH_3$,6-OH-pyrimidine treated mice, 67 % survival for 5-Cl-1,2,3-benzotriazole treated mice, 20 % survival for 2,6-di$NH_2$-9,10-anthraquinone treated mice, 14% survival for 1-$NH_2$,4-Cl-napthalene treated mice, and quite interestingly, 0 % survival for 4-Cl-acetylaniline treated mice even though the non-acetylated compound produced 68 % survival.

These substituted anilines represent an interesting class of radioprotectants. Their potential for influencing essential metalloelement metabolism in facilitating repair of radiation injury remains to be explained. Amino group sulfation or glucuronide formation offer potential pathways for the formation of ligands capable of acting as chelating agents in facilitating tissue distribution of essential metalloelements needed for *de novo* syntheses of essential metalloelement dependent enzymes required for repair of radiation injury.

**Radioprotectant Activity of Curcumin.**

Curcumin, a β-diketone {1,7-bis(4'-hydroxy-3'-methoxyphenyl)-1,6-heptadiene-3,5-dione}, is a yellow major component of Turmeric, a spice prepared from dried rhizomes of *Curcuma longa* L. Tumeric is widely used to season foods in Asian countries and in particular, Indian Curry. It is of particular interest to note that *Curcuma longa* L is a fungus and fungi are well known for their high copper content. Since β-diketones are well known chelating agents [123,124] and this fully conjugated β-diketone is likely to form particularly stable Cu chelates as a result of the extended conjugation in this ligand, it may be that this ligand exists as a Cu(II) chelate in fungi.

In 1993 Abraham, Sarma and Kesavan reported that Curcumin decreased chromosomal damage in γ-irradiated mice [125]. A single oral administration of 27.2 μmol Curcumin $kg^{-1}$ to male Swiss albino mice 2 hr **after** whole-body 1.15 Gy irradiation significantly (P = 0.01) reduced frequencies of micronucleated polychromatic erythrocytes (MPCEs) in bone marrow samples taken 24 hr after irradiation. Oral administration of 13.6, 27.2, or 54.4 μmol Curcumin $kg^{-1}$ 2 hr **before** irradiation produced a dose related decrease in MPCEs in bone marrow samples taken 24 hr after irradiation. Oral treatment with 27.2 μmol Curcumin $kg^{-1}$ 2 hr **before** irradiation produced significant (P = 0.05 to 0.01) time-dependent decreases in MPCEs in bone marrow samples taken 24, 30, and 48 hr after irradiation. These results suggest that Curcumin is useful in facilitating recovery from radiation injury.

Subsequently, Inano et al. [126] reported that feeding Curcumin decreased the incidence of mammary tumor in rats. A 1 % Curcumin diet fed to 2.6 Gy whole-body irradiated pregnant Wistar-MS rats just prior to pup birth (a mammary tumor initiating procedure) and then implanted with diethylstilbestrol sustained release pellets for 12 mo following weaning (a mammary tumor promoter regimen) caused a dramatically decreased incidence of mammary tumor. Tumor formation decreased from 84.6 % for the control diet fed group versus a 28 % incidence for the Curcumin fed rats. Feeding the diet containing Curcumin significantly (P<0.001) reduced the number of rats with tumors and the number of tumors per rat and increased the latency period for mammary tumor formation (P< 01001). Using this same pregnant Wistar-MS rat model of mammary tumorogenesis and feeding a 1 % Curcumin diet for a brief period from day-11 of pregnancy to day-23, the day of birth, with 1.5 Gy whole-body irradiation on day-20, prior to birth of the rat pups, again significantly

($P<0.0001$) decreased the number of dams with mammary tumors from 19 (70.3 % incidence) for the control group to 5 (18.5 % incidence) for the Curcumin fed rats. This Curcumin diet protocol also significantly ($P<0.0001$) delayed development of mammary tumors in Curcumin fed rats. Eventually mammary tumors did develop during the last two months of this 12-mo study [127]. Curcumin was fed for only 10 days in this study while it was fed for 1 yr in the first study (82). Thus further documenting and extending the application of dietary Curcumin in preventing irradiation-induced mammary tumors.

### CONCLUSIONS

Understanding essential metalloelement metabolism and its role in responding to radiation injury as mediated by immunomodulating cytokines does offer a renewed and potentially more effective approach to recovery from all radiation syndromes. Copper, Fe, Mn, and Zn chelates have radioprotectant activity based upon results obtained with treatment **before** radiation injury and some have been observed to have radiorecovery activity based upon results obtained with treatment **after** radiation injury. These compounds do not prevent radiation injury however they do facilitate repair and recovery from radiation injury. They are also far less toxic than existing radioprotectants. Existing radioprotectants have not been to shown to have radiorecovery activity. New chelates with increased efficacy and still less toxicity may be found using imaginative synthetic approaches to new ligands based upon existing compounds such as amino acids, salicylates, as well as radioprotectant aminothiols Ca-channel blockers, acyl Melatonin analogs, substituted anilines, and Curcumin. These chelates will also have potential for improving therapies of inflammatory diseases, ulcers, seizures, neoplastic diseases, diabetes, and pathology associated with ischemia-reperfusion

Combination treatment with essential metalloelement chelates and aminothiols, Ca-channel blockers, acyl Melatonin homologs, substituted anilines, and β-diketones such as Curcumin also offer an approach to improving radiation recovery and treatment of other recognized disease states. Combinations of some of these agents with essential metalloelement chelates may lead to the development of new physiological, biochemical, and pharmacological approaches to overcoming many disease states.

### ACKNOWLEDGEMENT

We are indebted to the National Institute for General Medical Sciences, PHS grant number S06-RR08211 for financial support.

### REFERENCES

[1] I.H. Tipton, M.J. Cook. *Health Phys.*, **1**, (1963) 103.

[2] G.V. Iyengar, W.E. Kollmer, J.M. Bowen. *The Elemental Composition of Human Tissues and Body Fluids*, Springer, New York, (1978).

[3] P.M. May, D.R. Williams. *FEBS Lett.* **78,** (1977) 134.

[4] J.R.J. Sorenson. Prog. Med. Chem. **26,** (1989) 437.

[5] E.L. Smith, R.L. Hill, I.R. Lehman, R.J. Lefkowitz, P. Handler, A. White. *Seventh Edition Principals of Biochemistry: General Aspects and Mammalian Biochemistry.* McGraw-Hill, New York, (1983).

[6] J.R. Prohaska. In *"Essential and Toxic Trace Elements in Human Health and Disease"*, (A.S. Prasad, Ed.); Alan R. Liss, New York, (1988) 105.

[7] R.E. Mains, B.A. Eipper, C.C. Glembotski, R.M. Dores. *Trends Neuro. Sci.* **6** (1983) 229.

[8] E. Weber, F.S. Esch, P. Bohlen, S. Paterson, A.D. Corbett, A.T. McKnight, H.W. Kosterlitz, J.D. Barchas, C.J. Evans. *Proc. Natl. Acad. Sci.* **80** (1983) 7362.

[9] D.C. Liebisch, B.R. Seizinger, G. Michael, A. Herz. *Neurochem.* **45** (1985) 1495.

[10] A. Bianchini, G. Musci, L. Calabrese. *J. Biol. Chem.* **274** (1999) 20265.

[11] J. Collinge, M.A. Whittington, K.C.L. Sidle, C.J. Smith, M.S. Palmer, A.R. Clarke, G.R. Jefferys. *Nature* **370** (1994) 295.

[12] T. Tamura, K.J. Hong, Y. Mizuno, K.E. Johnston, C.L. Keen. *Biochim. Biophys. Acta* **1427** (1999) 351.

[13] T. Borchardt, J. Camakaris, R. Cappai, C.L. Masters, K. Beyreuther, G. Multhaup. *Biochem. J.* **344** (1999) 461.

[14] K. Sekiya, H. Nagasaki, N. Ozaki, A. Suzuki, Y. Miura, Y. Oiso. *Biochem. Biophys. Res. Comm.* **278** (2000) 211.

[15] L. Rossi, M.R. Ciriolo, E. Marchese, A. De Martino, M. Giorgi, G. Rotilio. *Biochem. Biophys. Res. Comm.* **203** (1994) 1028.

[16] A.L. Lamb, A.S. Torres, T.V. O'Halloran, A.C. Rosenzweg. *Biochem.* **39** (2000) 14720.

[17] D.L. Huffman, T.V. O'Halloran. *J. Biol. Chem.* **275** (2000) 18611.
[18] A.L. Lamb, A.K. Wernimont, R.A. Pufahl, T.V. O'Halloran, A.C. Rosenzweig. *Biochem.* **39** (2000) 1589.
[19] S.X. Liu, J.P. Fabisiak, V.A. Tyurin, G.G. Borisenko, B.R. Pit, J.S. Lazo, V.E. Kagan. *Chem. Res. Toxicol.* **13** (2000) 922.
[20] A. Baba, T. Kihara, E. Lee, H. Iwata. *Biochem. Pharmacol.* **30** (1981) 171.
[21] A. White, K.M. Crawford, C.S. Patt, P.J. Lad. *J. Biol. Chem.* **251** (1976) 7304.
[22] R. Gerzer, E. Bohme, F. Hofmann, G. Schultz. *FEBS Lett.* **132** (1981) 71.
[23] O. Halevy, D.A. Sklan. *Life Sci.* **34** (1984) 1945.
[24] V.C. Culotta, T. Hsu, S. Hu, P. Furst, D. Hamer. *Proc. Natl. Acad. Sci.* **86** (1989) 8377.
[25] P. Klatt, K. Schmidt, G. Uray, B. Mayer. *J. Biol. Chem.* **268** (1993) 14781.
[26] G.D. Lawrence, D.T. Sawyer. *Coord. Chem. Revs.* **27** (1978) 173.
[27] M. Korc in "*Essential and Toxic Trace Elements in Human Health and Disease*". (A.S. Prasad, Ed.). Alan R. Liss, New York (1988) 253.
[28] V.L. Schramm, F.C. Wedler. *Manganese in Metabolism and Enzyme Function.* Academic Press, New York. 1986.
[29] E.J. Neer. *J. Biol. Chem.* **254** (1979) 2089.
[30] R.M. Leach,Jr. *Fed. Proced.* 30 (1971) 991.
[31] R. Myllyla, H. Anttinen, K.I. Kivirikko. *Euro. J. Biochem.* **101** (1979) 261.
[32] "*Metal Ions in Biological Systems: Manganese and its role in biological processes Volume 37.* (A. Sigel and H. Sigel. Eds.), Marcel Dekker, New York (2000)
[33] G. Westin, W. Shaffner. *Nucl. Acid Res.* **16** (1988) 5771.
[34] A. Klug, D. Rhodes. *Cold Spring Harbor Symp. in Quant. Biol.* **52** (1987) 473.
[35] L. Ravanti, V.M. Kahari. *Internat. J. Molec. Med.* **6** (2000) 391.
[36] A.A.D. Maur, T. Belser, Y. Wang, C. Gunes, P. Lichtlen, O. Georgiev, W. Schaffner, *Cell Stress Chaper.* **5** (2000) 196.
[37] L.M. Klevay, in "*Role of Copper in Lipid Metabolism.*" (K.Y. Lei, and T.P. Carr. Eds.), CRC Press, Boca Raton, (1990) 233.
[38] H.S. Wright, H.A. Guthrie, M.-Q. Wong, V. Bernardo. *Nutr. Today* **26** (1991) 21.
[39] J.R.J. Sorenson. *Inorg. Perspec. Biol. Med.*, **2** (1978) 1.
[40] M. Srivastava, R.K. Kale. *Rad. Res.* **152** (1999) 257.
[41] J.A. Fee, J.S. Valentine in "*Superoxide and Superoxide Dismutases.*" (A.M.;Michelson, J.M. McCord, I. Fridovich Eds.) Academic Press, New York (1977) 19.
[42] E.J. Corey, M. Mehrota, A.U. Khan. *Biochem. Biophys. Res. Comm.* **145** (1987) 842.
[43] K.N. Prasad in "*Radiation Biology.*" (D.J. Pizzarelli and L.G. Colombetti, Eds.) CRC Press, Boca Raton (1982) 205.
[44] D. Harrison, M. Ricciardello, L. Collins. *Aust NZ J. Med.* **28** (1998) 597.
[45] J.B. Little. *Carcinogenesis* **21** (2000) 397.
[46] J. Chatterjee, K. De, S.K. Basu, A.K. Das. *Indian J. Med. Res. [B]* **98** (1993) 243.
[47] A. Petrova, T. Gnedko, I. Maistrova, M. Zafranskaya, N. Dainiak. *Stem Cells* **15 (suppl 2)** (1997) 141.
[48] J.R.J., Sorenson, L.S.F., Soderberg, L.W. Chang. *Soc. Exp. Bio.l Med.* **210** (1995) 191.
[49] R.D. Henderson, T.D. Henderson, H.J. Irving, J.R.J. Sorenson. *Metal-Based Drugs* **6** (1999) 121.
[50] H.J. Irving, T.D. Henderson, R.D. Henderson, E.L. Williams, W.M. Willingham, J.R.J. Sorenson. *Inflammopharmacol.* **3** (1995) 251.
[51] M. Zhou, J.R.J. Sorenson. *J. Inorg. Biochem.* **72** (1998) 217.
[52 J.A. Kuykendall III, H. Simmons III, H.J. Irving, M.A. Wear, P.G. Sorenson, L.G. Tipton, K.M. Maddox, E.L. Williams, V.A. Pham-Tran, C. Credit, I.L. Chowdhury, S. Khan, J.B. Tipton, W.M. Willingham, J.R.J. Sorenson. *Metal-Based Drugs* **6** (1999) 135.
[53] D. Murry, W.H. McBride in "*Radioprotective agents. Kirk-Othmer Encyclopedia of Chemical Technology, Fourth Edition, Volume 20*" (1996) 963.
[54] R. Neta. Modulation of radiation damage by cytokines. *Stem Cells* **15 (suppl 2)** (1997) 87.
[55] C. Petit-Frère, E. Capulas, D.A. Lyon, C.J. Norbury, J.E. Lowe, P.H. Clingen, E. Riballo, M.H.L. Green, C.F. Arlett. *Carcinogenesis* **21** (2000) 1087.
[56] A.A.I. Daffada, S.P. Young. *FEBS Lett.* **457** (1999) 214.
[57] J.R.J. Sorenson. *Prog. Med. Chem.* **26** (1989) 437.

[58] M. Athar, H. Mukhtar, C.A. Elmets, M.T. Zaim, J.R. Lloyd, D.R. Bickers. *Biochem. Biophys. Res. Comm.* **151** (1988) 1054.
[59] M. Athar, C.A. Elmets, D.R. Bickers, H. Mukhtar. *J. Clin. Invest.* **83** (1989) 1137.
[60] K. Danno, T. Horio, M. Takigawa, S. Imamura. *J. Invest. Dermatol.* **83** (1984) 166.
[61] G. Deliconstantinos, V. Villiotou, C. Fassitsas. *J. Cardiovas. Pharmacol.* **20(suppl 12)** (1992) S63.
[62] Y. Miyachi, S. Imamuara, Y. Niwa. *J. Invest. Dermatol.* **89** (1987) 111.
[63] Y. Hashimoto, N. Ohkuma, H. Iizuka. *Arch. Dermatol. Res.* **283** (1991) 317.
[64] D.L. Bissett, R. Chatterjee, D.P. Hannon. *J. Soc. Cosmet. Chem.* **43** (1992) 85.
[65] A. Keshavarzian, J. Haydek, R. Zabihi, M. Doria, M. D'Astice, J.R.J. Sorenson. *Digest. Dis. Sci.* **37** (1992) 1866.
[66] J. Chatterjee, K. De, S.K. Basu, A.K. Das. *Indian J. Med. Res. [B]* **98** (1993) 243.
[67] J.-L. Lefaix, S. Delanian, J.-J. Leplat, Y. Tricaud, M. Martin, A. Nimrod, F. Baillet, F. Daburon. *Int. J. Radiat. Oncol. Biol. Phys.* **35** (1996) 305.
[68] J. Sun, Y. Chen, M. Li, Z. Ge. *Free Rad. Biol. Med.* **24** (1998) 586.
[69] T.V. O'Halloran in "*Metal Ions in Biological Systems. Metalloregulatory proteins: Metal-Responsive molecular switches governing gene expression.*" (H. Sigel and A. Sigel Eds.), New York, Marcel Dekker Inc. (1989) 105.
[70] G.S. Naeve, A.M. Vana, J.R. Eggold, G.S. Kelner, R. Maki, E.B. Desouza, A.C. Foster. *Neurosci.* **93** (1999) 1179.
[71] G.S. Kelner, M.-H. Lee, M.E. Clark, D. Maciejewski, D. McGrath, S. Rabizadeh, T. Lyons, D. Bredesen, P. Jenner, R.A. Maki. *J. Biol. Chem.* **275** (2000) 580.
[72] J.E. Swauger, P.M. Dolan, J.L. Zeier, P. Kuppusamy, T.W. Kensler. *Chem. Res. Toxicol.* **4** (1991) 223.
[73] C.J. Reed, K.T. Douglas. *Biochem. J.* **275** (1991) 601.
[74] A.S. Modak, J.K. Gard, M.C. Merriman, K.A. Winkler, J.K. Bashkin, M.K. Stern. *J. Am. Chem. Soc.* **113** (1991) 283.
[75] L.A. Basile, J.K. Barton. in "*Metal Ions in Biological Systems, Metallonucleases: Real and Artificial.*" (H. Sigel and A. Sigel, Eds.), New York, Marcel Dekker Inc., (1989) 31.
[76] S. Borah, M.S. Melvin, N. Lindquist. R.A. Manderville. *J. Am. Chem. Soc.* **120** (1998) 4557.
[77] J. Baquial, J.R.J. Sorenson. *J. Inorg. Biochem.* **47** (1992) 59.
[78] S.T. Shuff, P. Chowdhary, M.F. Khan, J.R.J. Sorenson. *Biochem. Pharmacol.* **43** (1992) 1601.
[79] F.T. Greenaway, J.J. Hahn, N. Xi, J.R.J. Sorenson. *Biometals* **11** (1998) 21.
[80] J.R.J. Sorenson. *J. Inorg. Biochem.* **43** (1991) 625.
[81] A.T. Gurbuz, J. Kunzelman, E.E. Ratzer. *J. Surg. Res.* **74** (1998) 149.
[82] J.G.L. Baquial, J.R.J. Sorenson. *Inorg. Biochem.* **60** (1995) 133.
[83] B.L. Booth, E. Pitters, B. Mayer, J.R.J. Sorenson. *Metal-Based Drugs* **6** (1999) 111.
[84] A. Bianchini, G. Musci, L. Calabrese. *J. Biol. Chem.* **274** (1999) 20265.
[85] D. Jourd'heuil, F.S. Laroux, D. Kang, A.M. Miles, D.A. Wink, M.B. Grisham. *Meth. Enzymol.* **301** (1999) 220.
[86] I.G. Kim, S.Y. Park. *FEBS Lett.* **437** (1998) 293.
[87] M.-K. Cha, I.-H. Kim. *Biochem.* **38** (1999) 12104.
[88] J.W. Tams, A.H. Johnsen, J. Fahrenkrug. *Biochem. J.* **341** (1999) 271.
[89] B.A. Eipper, D.A. Stoffers, R.E. Mains. *Ann. Rev. Neurosci.* **15** (1992) 57.
[90] K. Sekiya, H. Nagasaki, N. Ozaki, A. Suzuki. *Biochem. Biophys. Res. Comm.* **278** (2000) 211.
[91] H.J. Irving, M.A. Wear, H. Simmons, L.G. Tipton, J.B. Tipton, K.M. Maddox, W.M. Willingham, J.R.J. Sorenson. *Inflammopharmacol.* **4** (1996) 309.
[92] W.C. Hunt, R.A. Sorrenti, M.E. Renke, L.Y. Xie, E.M. Waterfield, J.G. Levy. *Immunopharmacol. Immunotoxicol.* **16** (1994) 55.
[93] R. Murata, Y. Nishimura, M. Hiraoka, M. Abe, M. Satoh. Manganese chloride treatment does not protect against acute radiation injury of skin or crypt cells. *Radiat. Res.* **143** (1995) 316.
[94] T.D. Henderson, R.D. Henderson, H.J. Irving, W.M. Willingham, J.R.J. Sorenson. *Inflammopharmacol.* **3** (1995) 241.
[95] V. Chhajlani, K.L. Axelsson, J. Ahlner, J.E.S. Wikberg. *Biochem. Internatl.* **19** (1989) 1039.
[96] B. Ortel, R.W. Gange, T. Hasan. *J. Photochem. B:Biol.* **7** (1990) 261.
[97] S.M. Hahn, A. Krishna, J.B. Mitchell, A. Russo. *Arch. Biochem. Biophys.* **288** (1991) 215.
[98] D.J. Darr, S. Yanni, S.R. Pinnell. *Free Rad. Biol. Med.* **4** (1988) 357.
[99] D. Darr, K.A. Zarilla, I. Fridovich. *Arch. Biochem. Biophys.* **258** (1987) 351.

[100] J.S.L. Mok, K. Paisley, W. Martin. *Brit. J. Pharmacol.* **124** (1998) 111.
[101] J.A. Kuykendall III, R.H. Ebert II, I.H. El-Sayed, P.K. Roberson, V.A. Pham-Tran, B.L. Booth, T. Brooks, W.M. Willingham, J.R.J. Sorenson. *Metal-Based Drugs* **6** (1999) 127.
[102] M.W. Epperly, J.A. Gretton, S.J. DeFilippi, C.A. Sikora, D. Liggit, G. Koe, J.S. Greenberger. *Rad. Res.* **155** (2001) 2.
[103] M.W. Epperly, E.L. Travis, J.A. Whitsett, I. Raineri, C.J. Epstein, J.S. Greenberger. *Internat. J. Cancer* **96** (2001) 11.
[104] A.N. Osipov, V.D. Sypin, G.Ya. Kolomijtseva. *Biochem (Moscow)* **64** (1999) 201.
[105] M.-T. Leccia, M.-J. Richard, A. Favier, J.C. Béani. *Biol. Trace Elem. Res.* **69** (1999) 177.
[106] J. Mathieu, S. Ferlat, D. Ferrand, B. Ballester, S. Platel, V. Gerard, Y. Cancerella, J.-C. Mestries, J.-F. Kergonou. *Biochem. Mol. Biol. Internat.* **36** (1995) 733.
[107] G.L. Floersheim, N. Chiodetti, A. Bieri. *Brit. .J. Radiol.* **61** (1988) 501.
[108] G.L. Floersheim, A. Bieri. *Brit. J. Radiol.* **63** (1990) 468.
[109] G.L. Floersheim, A. Christ, R. Koening, C. Racine, F. Gudat. *Int. J. Cancer* **52** (1992) 604.
[110] J.M. Berg in *"Metal Ions in Biological Systems. Zinc Fingers: The role of zinc(II) in transcription factor III A and related proteins."* (H. Sigel and A. Sigel, Eds.), New York, Marcel Dekker Inc. (1989) 235.
[111] Y. Samuni, D. Coffin, A.M. DeLuca, W.G. DeGraff, D.J. Venson, I. Ambudkar, M. Chevion, J.B. Mitchell. *Cancer Res.* **59** (1999) 405.
[112] T. Ushakova, H. Melkonyan, L. Nikonova, V. Afanasyev, A.I. Gaziev, N. Mudrik, R. Bradbury, V. Gogvadze. *Free Rad. Biol. Med.* **26** (1999) 887.
[113] G.L. Floersheim. *Brit. J. Radiol.* **65** (1992) 1025.
[114] G.L. Floersheim. *Radiat. Res.* **133** (1993) 80.
[115] H.J. Koch. *Radiat. Res.* **135** (1993) 438.
[116] G.L. Floersheim. *Radiat. Res.* **135** (1993) 439.
[117] T. Elnimr, S.M. Abdel-Rahim. *Biol. Trace Element. Res.* **62** (1998) 25.
[118] M. Karbownik, R.J. Reiter. *Proced. Soc. Exptl. Biol. Med.* **225** (2000) 9.
[119] R.T. Blickenstaff, S.M. Brandstadter, S. Reddy, R. Witt. *J. Pharm. Sci.* **83** (1994) 216.
[120] Vijayalaxmi, M.L. Meltz, R.J. Reiter, T.S. Herman, K.S. Kumar. *Mutat. Res.* **425** (1999) 21.
[121] M. Karbownik, R.J. Reiter, W. Qi, J.J. Garcia, D.-T. Tan, L.C. Manchester, Vijayalaxmi. *Mol. Cell. Biochem.* **211** (2000) 137.
[122] R.T. Blickenstaff, S.M. Brandstadter, S. Reddy, R. Witt, K.B. Lipkowitz. *J. Pharm. Sci.* **83** (1994) 219.
[123] K, Schwarz, W. Mertz. *Fed. Proceed.* **20 Suppl 10** (1961) 111.
[124] A. Albert. *Fed. Proceed.* **20 Suppl 10** (1961) 137.
[125] S.K. Abraham, L. Sarma, P.C. Kesavan. *Mutat. Res.* **303** (1993) 109.
[126] H. Inano, M. Onoda, N. Inafuku, M. Kubota, Y. Kamada, T. Osawa, H. Kobayashi, K. Wakabayashi. *Carcinogenesis* **20** (1999) 1011.
[127] H. Inano, M. Onoda, N. Inafuku, M. Kubota, Y. Kamada, T. Osawa, H. Kobayashi, K. Wakabayashi. *Carcinogenesis* **21** (2000) 1835.

**Received: December 7, 2001 – Accepted: December 20, 2001 – Accepted in publishable format: December 21, 2001**

